# Expanding Fortification with Folic Acid: Thinking Outside the Cereal-Grain Box

**DOI:** 10.3390/nu16091312

**Published:** 2024-04-27

**Authors:** Becky L. Tsang, Carlen Stadnik, Michelle Duong, Helena Pachón, Homero Martinez

**Affiliations:** 1 Food Fortification Initiative, Atlanta, GA 30322, USA; carlenstadnik@gmail.com (C.S.); mduong4@alumni.emory.edu (M.D.); helena.pachon@emory.edu (H.P.); 2 Rollins School of Public Health, Emory University, Atlanta, GA 30322, USA; 3 Nutrition International, Ottawa, ON K2P 2K3, Canada; hmartinez@nutritionintl.org; 4 Hospital Infantil de Mexico Federico Gomez, Mexico City 06720, Mexico

**Keywords:** folic acid, fortification, neural tube defects

## Abstract

(1) Background: Fortifying maize and wheat flours with folic acid has effectively reduced neural tube defect-affected births. However, maize and wheat flours may not be widely consumed in all countries; further reduction in neural tube defect-affected births could benefit from the identification of alternative food vehicles. We aimed to use dietary intake or apparent consumption data to determine alternative food vehicles for large-scale fortification with folic acid in low-income and lower-middle-income countries (LILMICs) and identify current research related to examining the technological feasibility of fortifying alternative foods with folic acid. (2) Methods: We identified 81 LILMICs, defined by the World Bank’s (WB) 2018 income classifications. To identify dietary intake or apparent consumption, we reviewed WB’s Microdata Library and Global Health Data Exchange for national surveys from 1997–2018. We reviewed survey reports for dietary intake or apparent consumption data and analyzed survey datasets for population coverage of foods. We defined alternative food vehicles as those that may cover/be consumed by ≥30% of the population or households; cereal grains (maize and wheat flours and rice) were included as an alternative food vehicle if a country did not have existing mandatory fortification legislation. To identify current research on fortification with folic acid in foods other than cereal grains, we conducted a systematic review of published literature and unpublished theses, and screened for foods or food products. (3) Results: We extracted or analyzed data from 18 national surveys and countries. The alternative foods most represented in the surveys were oil (*n* = 16), sugar (*n* = 16), and salt (*n* = 14). The coverage of oil ranged from 33.2 to 95.7%, sugar from 32.2 to 98.4%, and salt from 49.8 to 99.9%. We found 34 eligible studies describing research on alternative foods. The most studied alternative foods for fortification with folic acid were dairy products (*n* = 10), salt (*n* = 6), and various fruit juices (*n* = 5). (4) Conclusions: Because of their high coverage, oil, sugar, and salt emerge as potential alternative foods for large-scale fortification with folic acid. However, except for salt, there are limited or no studies examining the technological feasibility of fortifying these foods with folic acid.

## 1. Introduction

Food fortification is the practice of adding essential vitamins and minerals to foods at the post-harvest stage (e.g., food milling and processing) to improve dietary intake [1]. Salt was one of the first foods in the world to be fortified in the 1920s in Switzerland with iodine [2] to treat and prevent iodine deficiency disorders that lead to developmental disabilities. Wheat flour fortification was initiated in the UK in 1940 and later introduced in the United States due to the poor nutritional status of potential recruits during World War II [3].

After the studies in the 1990s [4,5,6] collectively demonstrated that folic acid (the synthetic form of folate [vitamin B9]) supplementation in women during the periconceptional period prevented a majority of severe birth defects of the brain and spine called neural tube defects (NTD), folic acid was incorporated into existing wheat flour fortification programs in many countries. Oman was the first country to do so in 1996 [7], with the United States and Canada closely following in 1998 [8]. As of May 2022, 91 countries mandate the fortification of maize flour, rice, or wheat flour with vitamins and minerals, and of those, 67 countries include folic acid as a mandatory nutrient in fortification standards [9].

Several countries have reported lower NTD rates in the post-fortification period compared to the pre-fortification period [10], indicating that food fortification with folic acid translates into a similar biological impact as supplementation with folic acid, but with a wider population coverage.

However, since it began in 1996, fortification with folic acid on a widespread, public health scale has been limited to maize flour, rice, and wheat flour. While these three cereal grains are staple foods for more than half of the population in the world [11], there are still many countries and subpopulations where fortified or industrially milled (fortifiable) cereal grains may not be the optimal food vehicle for fortification due to dietary customs and/or lack of modernized milling industries. In these countries and/or subpopulations, alternative foods (if industrially processed) with adequate population coverage may be better suited to improve the dietary intake of folic acid to prevent NTDs. There are already efforts to explore the opportunity of fortifying alternative foods with folic acid, for example, iodized salt with folic acid in Ethiopia [12].

With this in mind, our objectives were to (1) analyze national surveys of food intake (via dietary surveys) or apparent consumption (via household income and expenditure surveys) to identify potential alternative food vehicles in low-income and lower-middle-income countries (LIMICs) and (2) conduct a systematic review to describe the available research around the efficacy, retention, food characteristics, and acceptability of fortifying alternative foods (other than maize and wheat flours and rice) with folic acid to identify knowledge gaps.

## 2. Subjects and Methods

We did not seek review from an institutional ethical review board because we used existing, de-identified datasets in our analysis.

### 2.1. Objective 1: Identifying Foods with ≥30% Coverage in LILMICs

To identify the coverage of specific foods, we analyzed nationally representative surveys. Dietary consumption survey data, reflecting reported consumption of specific foods (not food groups or categories of foods), were considered eligible, as were household income and expenditure data, which provide a proxy for consumption through reported purchases (including food items) at the household level (referred to as apparent consumption). To be as inclusive as possible, we conservatively defined adequate coverage as ≥30%.

#### 2.1.1. Data Sources

Two databases for country surveys were utilized to identify eligible data: the World Bank’s Microdata Library, which contains references to country surveys and reports that include living standards and/or economic indicators, and the Institute of Health Metrics and Evaluation’s Global Health Data Exchange (GHDx), which contains references to health and nutrition surveys and reports that include nutrition indicators.

#### 2.1.2. Screening Process to Identify Potential Eligible Surveys (Figure 1)

Eighty-one countries classified by the World Bank in 2018 as LILMICs were eligible. We conducted the search in January 2019; as such, search results were filtered to include surveys conducted/published between 1997 and 2018. Country survey titles were reviewed to assess whether food consumption or expenditure data were included. Terms used to define surveys as likely eligible included “living standards”, “household income”, “nutrition”, “micronutrient”, “consumption”, and “household survey”. Both the World Bank and GHDx sites include Demographic Health Surveys and Multiple Indicator Cluster Surveys; however, these survey titles were not considered eligible as they are usually standardized survey tools (modified as needed per country needs) that do not include food consumption or apparent consumption as defined by us.

**Figure 1:**
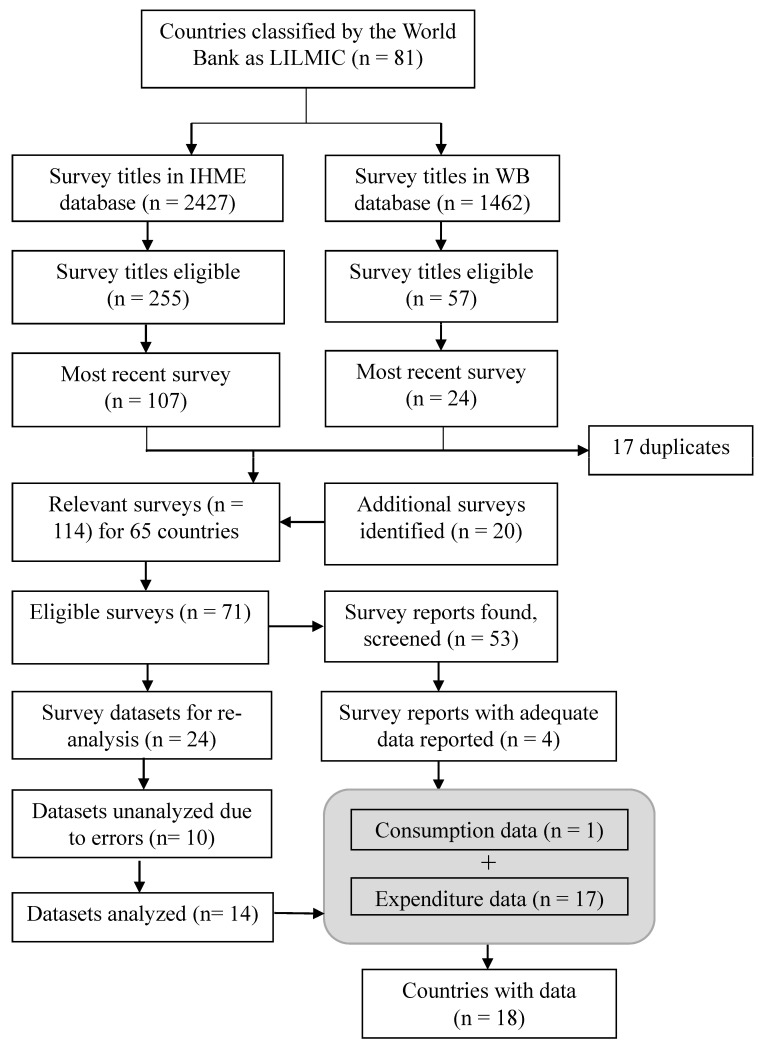
Flow diagram for identifying national surveys with dietary intake, household expenditure/consumption, and/or micronutrient status data. The grey box represents the combination of surveys to represent the number of countries with eligible data. FFI, Food Fortification Initiative; IHME, Institute for Health Metrics and Evaluation; LILMICs, low-income and lower-middle-income countries; WB, World Bank.

The GHDx database also provided alternative titles which may have been in another language, or a secondary survey name. These alternative titles were also screened for eligibility. Subnational data in the GHDx database were excluded. After a complete list of all surveys with potential consumption or expenditure data was identified, we narrowed down the search for survey documents to only the most recent survey year of any given survey series for each country (e.g., we only searched for survey documents and reports for a 2015 Household Expenditure Survey, even if a 2010 survey existed). Surveys were only considered part of a survey series if the names matched exactly; otherwise, they were considered independent surveys, and eligible for further review.

The eligible survey titles from the World Bank and GHDx were merged to remove any duplicates that may have been found in both databases. Eligible survey titles (in English and the country’s official language, if not English) were entered into Google to collect as many supporting documents as possible—including data dictionaries, final reports, meta-data, survey tools (questionnaires), and datasets. These materials were often available in the World Bank’s Microdata Central Library; GHDx does not provide supporting survey material in its database. In some cases, Food Fortification Initiative (FFI) staff supporting national fortification programs in specific countries identified additional surveys or survey materials not found online.

#### 2.1.3. Screening Process to Confirm Eligible Surveys

Each survey and its supporting documents were screened to check whether the surveys collected and/or reported food intake or apparent consumption data. If a report was available, it was reviewed to check whether data on food consumption or expenditure were sufficiently reported for the objective’s needs (e.g., a listing of all foods reported consumed or purchased, not a list of food categories or minimum diet quality). Reports in languages other than English were reviewed in Google Translate for eligibility. If the report was in Spanish, Portuguese, or French, co-authors or colleagues provided support to double-check its eligibility. If the information in the report was sufficient, the data were extracted as reported. If no report was found, or the data in the report were inadequately reported, a dataset was sought for further analysis.

Datasets were identified through three channels: direct download from the World Bank Microdata Library, direct download from a nationally operated microdata library, and emailed requests to national statistics organizations if survey contacts or responsible organizations for the organization could be readily identified, which may have included completing data license forms.

#### 2.1.4. Data Analysis

If a dataset was available for analysis, then analyses were conducted in SAS (SAS 9.4, Cary, NC, USA) to report the proportion of households or individuals purchasing/consuming a particular food, primarily using PROC FREQ or PROC SURVEYFREQ (if any survey weighting or clustering data were available). If possible, adjusted analyses were conducted to take into account survey weighting. If datasets were in a language other than English, Google Translate was used to translate analysis results (except where the co-authors assisted with Spanish, Portuguese, and French).

#### 2.1.5. Identifying Foods as Potential Alternative Vehicles

A food was considered a ‘potential alternative vehicle’ for folic acid if there was no existing mandatory fortification with folic acid, and there are existing large-scale efforts in food fortification (not just with folic acid), i.e., any cereal grain, oil, sugar, condiment, or milk. While many countries may have mandatory salt and/or oil fortification, there are no countries that require the addition of folic acid to either food [13]. As such, these foods are considered alternative vehicles for folic acid. Condiments were only considered potential alternative vehicles if the food listed was specific (e.g., bouillon cubes), and not under an umbrella category (e.g., spices, condiments, sauces). Except for wheat flour, derivatives of maize flour, rice, oil, sugar, condiments, or milk were not flagged as potential alternative vehicles (e.g., candy products were not flagged as potential alternative vehicles because we considered them a sugar derivative). This exception was made for wheat flour because food lists in household income and expenditure surveys frequently reference the wheat flour product in addition/instead of wheat flour itself.

### 2.2. Objective 2: Systematic Review of Research on Fortifying Alternative Foods with Folic Acid

The protocol for the systematic review is available in the Supplementary Materials Document. We defined eligible foods as a food that already exists; it did not refer to a food that was newly created for the purposes of fortification. We included occasions where researchers may have used two distinct foods and combined them to make a fortified food (e.g., the combination of milk and orange juice to make another beverage). The food did not have to be explicitly described (e.g., a description of a ‘beverage’ without any other identifying information was acceptable). We used Covidence [14] to screen studies for eligibility at the title/abstract and full-text stage.

#### 2.2.1. Search Strategy

In November 2018, we searched the following databases: Systematic Review of Agricultural & Environmental Science Database, CAB Abstracts, EBSCO, EMBASE, ProQuest Dissertations and Theses Global, PubMed, Scopus (SciVerse Scopus), and Web of Science for Medical Subject Headings in PubMed. The search terms were the following; the terms were modified for each database: (fortify OR fortifies OR fortified OR fortifying OR fortification OR enrich OR enriches OR enriched OR enriching OR enrichment OR enriched food OR enriched foods OR fortified food OR fortified foods OR functional food OR functional foods) AND (folic acid OR folate OR vitamin B9) AND (salt OR salts OR sugar OR sugars OR fat OR fats OR oil OR oils OR butter OR butters OR margarine OR margarines OR beverage OR beverages OR water OR juice OR juices OR milk OR condiment OR condiments OR soup OR soups OR cube OR cubes OR bouillon OR bouillons OR sauce OR sauces OR paste OR pastes OR new OR novel OR potential OR possible OR possibility OR possibilities OR suitable OR suitability OR acceptable OR acceptability).

#### 2.2.2. Inclusion Criteria

We included stability/retention studies with at least two time points, studies describing a food’s characteristics after fortification with folic acid (e.g., consumer panel taste tests, preference ratings, color or texture assessments, and other food characteristic tests as defined by authors), and efficacy studies (e.g., controlled feeding trials with at least two time points). Efficacy studies were only eligible if the effect of folic acid could be isolated; i.e., appropriate comparisons were a folic acid-fortified food vs. placebo or multiple micronutrients with folic acid vs. multiple micronutrients without folic acid. Thus, if the food was fortified with multiple micronutrients (in addition to folic acid) and the outcomes could not be attributed to the addition of folic acid, the study was not accepted for final eligibility (for efficacy outcomes). However, in consideration that certain efficacy outcomes could be considered specific to folate, we maintained a reference list of these eliminated studies (Supplementary Table S10).

For those studies that included human participants, we included females and males (any age), regardless of baseline anemia or folate status. Folic acid supplementation through animal feed was acceptable if the purpose was to improve human health; such studies had to include outcomes measured in humans. For example, a study aiming to increase the folic acid content of eggs through folic acid-fortified chicken feed would only have been eligible if it also assessed folate status in humans consuming the resulting eggs. Any research published during or after 1980—around the time when folate’s role in preventing NTDs was proposed by Smithells et al. [15]—was included.

#### 2.2.3. Exclusion Criteria

We excluded animal studies unless the study involved animal feeding to improve the folic acid content of an alternative food and assessed its impact on human health. Given our focus on alternative foods for the fortification with folic acid, we did not include research on the fortification of wheat flour, maize flour, or rice (or their derivatives, such as cookies, biscuits, and noodles made with wheat flour, maize flour, or rice/rice flour) with folic acid; other grains were acceptable. Supplemental/complementary foods, defined as foods with a therapeutic nutrition purpose, point-of-use fortification (e.g., folic acid added to food at home or a feeding site just before consumption), foods fortified through folate production by bacteria, and biofortified foods were excluded. In vitro, bioaccessibility models that determined folate bioavailability were also excluded.

Given the broad set of study outcomes eligible for the review, we did not conduct a quality assessment of the studies included in our descriptive analysis.

#### 2.2.4. Data Extraction and Management

Data extraction was conducted independently and in duplicate using a digital extraction form piloted and designed for this review. Data from the following domains were collected: study methodology (study design, unit of randomization, participant selection, method of allocation, sample size, assessment methods for each outcome) and participant details (study location, age, health status, baseline anemia and nutritional status, author-defined inclusion/exclusion criteria). For efficacy studies, we also extracted information on the intervention/exposure: folic acid dose, fortification method, food vehicle, duration of the intervention, co-interventions, and comparison group(s). For stability/retention studies, we extracted pre/post measurements, retention estimates during various points in the food product’s supply chain (production, storage, and cooking; conditions during production and storage that may affect the stability of the nutrient, e.g., light, temperature, humidity, packaging, as reported by authors), and food characteristics (taste, smell, appearance, texture, and other factors as described by authors).

## 3. Results

### 3.1. Objective 1: Identifying Foods with >30% Coverage in LILMIC

The survey titles screened and ultimately identified as eligible are shown in Figure 1. Starting with 81 eligible LILMICs, 3889 survey titles relevant to these countries from the GHDx and World Bank databases were screened. At all stages, it was possible for countries to have multiple surveys with potential data. As such, Figure 1 distinguishes between the number of surveys/reports at each screening stage (where there may be duplicate countries) versus the number of countries. Of 18 countries identified with data, 17 surveys were based on food expenditure (i.e., apparent consumption) rather than consumption surveys using dietary intake tools (e.g., 24 h recalls or food frequency questionnaires). The Philippines was the only exception, reporting dietary intake from a 24 h recall [16].

Table 1 provides an overview of all the surveys (18 countries) identified in Figure 1. Of the eighteen countries, two are in the Latin America and Caribbean region, two in the Europe and Central Asia region, one in the South Asia region, and one in the East Pacific and Asia region; the remaining are in the Sub-Saharan Africa region. Although the autonomous region of Somaliland is not recognized as an independent country by the World Bank, a living standards survey had been conducted by the World Bank and was thus included in our results.

Table 2 collates the alternative food vehicles with coverage of >30% by number of occurrences across the 18 countries and identifies each country where it was an opportunity. Across these countries, oil (*n* = 16), sugar (*n* = 16), salt (*n* = 14), and rice (*n* = 12) had coverage of >30% in more than half of the countries and represent the four foods that we identified as offering the most opportunities. The only liquid food vehicle identified was milk (*n* = 6). Following these foods, grains without existing folic acid fortification were identified, including wheat flour (*n* = 7), maize flour (*n* = 3), millet (*n* = 3), sorghum (*n* = 2), and teff (*n* = 1). Bouillon cubes (*n* = 3) were the only identified condiment with multiple ingredients.

For detailed food coverage by country, Supplementary Table S1a–q provides lists of foods with >30% coverage by country (data from Uganda are not shown due to irregularities and suspected poor data quality; of the 76 foods queried in Uganda, all except one had 95% or greater coverage). Each food is described as-is by the survey itself, which led to food descriptions that are not consistent across surveys (e.g., edible oil vs. processed oils). Foods that do not have a direct translation into English are described using the local word/phrase for that food.

The screening results identified several data gaps and opportunities. Supplementary Table S2 provides a list of national surveys where screening found that there were consumption or food expenditure data available; however, datasets were not available for analysis, or data were not sufficiently detailed in a report. Supplementary Table S3 provides a list of countries where we could not verify whether the survey had consumption or food expenditure data—a report, survey tool, or dataset was not available to identify the methods used in the survey. Supplementary Table S4 lists LILMICs where screening did not identify any likely national surveys, whether known or unverified, that had consumption or food expenditure data.

### 3.2. Objective 2: Systematic Review of Research on Fortifying Alternative Foods with Folic Acid

**Figure 2:**
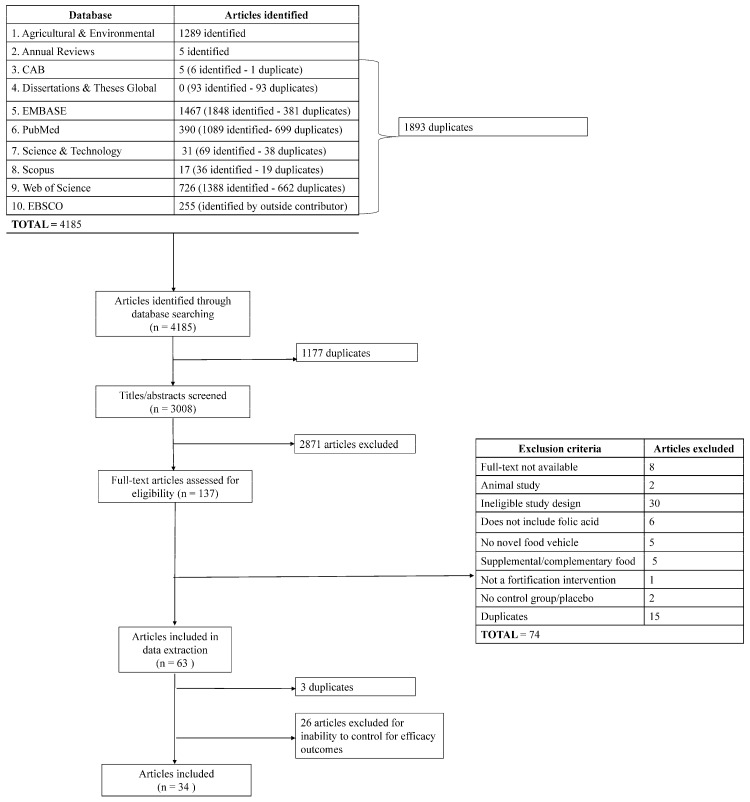
PRISMA flow diagram. PRISMA, Preferred Reporting Items for Systematic Reviews and Meta-Analyses.

Figure 2 describes the selection flow of literature at each screening stage. A total of 34 articles were included in the final data extraction tables (Table 3). Table 3 provides an overview of all included articles. Supplementary Tables S5–S9 present the research results by food category (dairy, condiments and spreads, beverages, meat and eggs, fruit, non-maize/rice/wheat cereal grains, and candy). One study [34] included two food categories (dairy and beverages) and, as a result, appears in both food categories. Other studies that included multiple foods assessed another food in the same category (e.g., salt and sugar, both categorized as condiments).

Supplementary Table S10 presents the list of efficacy studies eliminated due to multi-micronutrient fortification without an appropriate control food that would allow us to attribute efficacy to folic acid. However, where those studies assessed a blood folate outcome (i.e., serum/plasma and/or red blood cell folate, considered folate-specific), the outcome was bolded.

#### 3.2.1. Breadth of Study Outcomes

The dairy category (specifically milk) had the most varied study outcomes, encompassing retention during various stages of food production (e.g., pasteurization), storage, efficacy, and consumer acceptability. An outcome that was not assessed was the impact on folic acid after further food processing into a milk derivative (e.g., yogurt or cheese). Studies that did assess milk derivatives conducted fortification at that food’s production stage, but did not use a base of fortified milk (e.g., fortifying yogurt, instead of using fortified milk to produce the yogurt).

In some cases, several foods were studied exclusively by one or two research group(s) (e.g., University of Toronto for iodine/folic acid-fortified salt [47,48,49,50]; Sundar Serendipity, for multi-nutrient fortified salt [45,46]; and Galán et al. for meat products [59,60,61,62]).

##### Study Outcomes: Efficacy

Efficacy outcomes were limited to four studies on milk [35,37,38,39] and one on a low-fat spread (no other product details described [44]) (Supplementary Table S5). In all five studies, there were statistically significant increases in blood folate outcomes (serum/plasma folate, red blood cell folate) after consuming the fortified food. Two studies included homocysteine concentrations; homocysteine levels also consistently fell after consumption of a fortified food [38,39]. These results are consistent with folic acid efficacy studies in existing staple foods [68] and indicate that there is nothing different in the food matrixes of milk and a low-fat spread that would change how the body absorbs folic acid.

##### Study Outcomes: Retention

The retention of folic acid (alternatively described as stability) can be studied at several stages of food production, depending on the food in question. Studies included evaluated folic acid retention after heat treatment during a food’s production process (e.g., pasteurization in the case of milk and yogurt, irradiation in the case of meat products), as well as retention after a period of storage (Supplementary Table S5). Storage conditions varied by food studied, depending on the researchers’ expectations for a food’s storage conditions in field settings (e.g., refrigerated conditions for milk, high heat and humidity for salt). The length of storage time also varied, from six days to 20 months.

Generally, researchers described high folic acid retention in the varied conditions that were studied, with several stating no statistically significant or <10% losses after storage or food preparation/processing [34,36,45,46,47,51,52,57,58]. The highest folic acid losses occurred when salt was co-fortified with iodine and iron (30–40% losses [49]), after certain treatment processes (e.g., irradiation of meat, 20–30% losses [59,60,61]; addition of ethanol, 10–45% losses [55]) and in certain juices/fruit products [34,53,54]. However, the losses ranged widely depending on other food processing treatments that the juices underwent. Encapsulated folic acid, not currently used in cereal grain fortification, improved nutrient retention in orange or apple juice compared to the same juice with non-encapsulated folic acid, depending again on treatment processes and storage [53]. One study reported that losses occurred in the initial 15 days and plateaued after that [52]. Broadly, folic acid retention across various studied foods was favorable (i.e., above 50% retention) [69,70].

##### Study Outcomes: Food Characteristics

As fortification with a nutrient can potentially affect a food’s characteristics beyond nutrient content (e.g., color, as with the use of certain iron compounds in rice fortification [71]), it is also relevant to consider whether adding folic acid to a food will affect the quality of the food. Unlike iron, only one folate compound is used in fortification—folic acid. While studies (not found in this review) have evaluated the bioavailability of 5-methyl tetrahydrofolate [72] to assess the suitability of using a folate compound with better bioavailability by individuals with the methylenetetrahydrofolate reductase 677/TT genotype, this folate compound is not used in fortification due to its heat sensitivity. This greatly simplifies research in fortifying foods with folic acid, as there is no need to test for outcomes across multiple compounds.

Food characteristics and their relative importance in fortification will naturally depend on the type of food being fortified. Color may be a less important characteristic if the food is dark in color, compared to a food that is usually white, as demonstrated by Galán et al. in multiple studies that found adding folic acid visually acceptable in various meat products [59,60,61,62]. Flavor or color also may be less important if the food will eventually be flavored, as demonstrated by Boeneke et al., who found lemon-flavored yogurt fortified with folic acid rated higher on flavor than unflavored yogurt fortified with folic acid [41] (there was no comparison to non-fortified lemon-flavored yogurt).

Across studies, the most commonly assessed food characteristics were related to color, pH, texture (e.g., viscosity, shear force), and, in some cases, fat and protein. The research was heavily dominated by the effects on food characteristics in dairy, but in general, the studies consistently found little effect due to the addition of folic acid—with the exception of color. Folic acid is a bright yellow powder substance; several studies assessing color through the L*a*b* color space found increased values for b*, which measures the “yellowness” of a color. All studies assessing color using the L*a*b* color space found increased b* values [36,40,41,42,61] and/or visibly different food products [43,65], even though fortification levels varied by product. Results in salt were mixed, with descriptive reports of iodine and folic acid-fortified salt that was initially a pale yellow color but became pinkish after 20 months of storage at 45 °C and fluctuating humidity [50], and samples that were not visibly different in appearance even after nine months of storage in 60% relative humidity [48]. Food characteristics were not measured in beverages.

##### Study Outcomes: Consumer Acceptability

Studies used a Likert or hedonic scale to conduct consumer panels and assess the acceptability of the fortified food after fortification, usually against several sensory features (e.g., appearance, smell, taste, texture). However, methods differed, as some studies assessed acceptability after a period of storage of a raw food vs. cooked food, and not all studies provided a non-fortified control to compare acceptability against. In the limited number of foods where consumer acceptability was conducted (milk [36,43], meat products [59,60,61,62], fruit-based products [63,64]), there was high reported acceptability of the product after fortification, except in the case of unflavored, fat-free fortified yogurt, where increased acidity and effects in texture attributed to folic acid were reported as less acceptable to panelists [40]. There were no studies on the acceptability of beverages or condiments.

## 4. Discussion

We aimed to identify alternative food vehicles that could be opportunities for fortification with folic acid and to pair that with a summary of the research landscape for novel foods. Foods with adequate coverage (as defined by ≥30% of the population consuming that food) according to currently available surveys and data are (with at least six occurrences in the most consumed foods in that population, in descending order) oil, sugar, salt, rice, wheat flour, and milk (both liquid and powder). The current evidence for fortifying ‘alternative’ foods with folic acid is in poor alignment with the foods having the highest coverage across the countries/populations with data available. Most research on fortifying foods with folic acid was conducted in dairy products (efficacy, retention, food characteristics), while no studies were found on fortifying oil with folic acid and only one study on the fortification of sugar with folic acid (retention and food characteristics). Although milk could potentially be an alternative food vehicle in countries where coverage is adequate, the available research was limited to efficacy, with only a limited understanding of how milk characteristics (e.g., color) may be affected, and no consumer acceptability studies. Studies on the fortification of salt with folic acid are largely limited to retention studies, with mixed results regarding sensory effects and little attention to efficacy or downstream effects on foods prepared with folic acid-fortified salt. Efforts to fill in such data gaps are already underway to examine the acceptance and efficacy of folic acid-fortified salt in Ethiopia [73]. In a total of 100 countries which had data on the percentage of households consuming iodized salt, a study modeled that 180,000 cases of spina bifida and anencephaly could be prevented through folic acid-fortified iodized salt, and 150,000 of these 180,000 cases are occurring in countries where more than 80% households consume iodized salt [74].

Our work provides the fortification community with a clearer view of the foods with adequate coverage across multiple countries and dietary patterns and the evidence gaps that exist for these foods. This information can be used for developing a strategy for expanding the prevention of neural tube defects through fortification, by avoiding duplication where there is already adequate evidence, verifying where there is conflicting research, and filling in implementation and feasibility gaps needed for engaging with national decision-makers. Strengths of this work include a systematic review process for both the identification of national surveys and research literature, the use of proxy data (apparent consumption) where dietary intake data were unavailable, and broad eligibility of research outcomes.

### 4.1. Objective 1: Identifying Foods with >30% Coverage in LILMICs

#### 4.1.1. Data Quality

With the exception of the Philippines, all data to describe potential new food vehicles came from household income and expenditure surveys. There are benefits but also limitations to using apparent consumption as a proxy measurement for consumption [75], the most notable limitation being that household purchases of foods may not accurately reflect the consumption of foods prepared or purchased outside of the home. This may impact the accuracy of describing the coverage of specific food items and/or ingredients that are more likely to be consumed outside of the home (e.g., packaged foods or meals prepared outside the home).

Household income and expenditure data are also, by definition, specific to the household, not to an individual person in that household. Thus, it is not possible to describe the coverage of a food for target groups of interest within that household (e.g., women of reproductive age). With household income and expenditure data, an assumption was made that household purchases are consumed by the entire household. Although we did not consider food consumption amounts as criteria for a fortification vehicle, another limitation of apparent consumption is that assumptions, such as applying an adult male equivalent value, must be made about the amount of food that is consumed by different target groups of interest [76].

In addition, depending on the tool used, household income and expenditure surveys are often not a comprehensive recounting of all foods a household consumes (unlike a 24 h recall)—usually, these surveys refer to a specific list of pre-identified, commonly consumed foods that should be tailored to the cultural context of that country. Surveys without appropriately adapted food lists will have inaccurate descriptions of foods most commonly purchased by the household [77]. Survey instruments may also vary by recall period—e.g., apparent consumption in the last 7 days versus in the last 30 days. Surveys with a longer eligible time period could overestimate a food’s potential coverage in the population, as the variety of foods a household eats within 30 days is likely more varied than in the last 7 days and could include foods that are eaten with low frequency; conversely, food coverage could be underestimated if not all foods are recalled during the longer period.

The consumer acceptability studies within our review should be considered with caution, as the participants received the food for free in a study setting. It could be argued that such participants are more discerning towards a product that they are expected to self-purchase (especially if competing less expensive, non-fortified products are available in the marketplace). The included studies also ranged widely in methodology; when/how measurements of acceptability should be conducted will depend on the food and how it is typically eaten by the consumer. In the case of dry, fermented sausages, if they are typically consumed after 90 days of aging, then it will be more important to understand if folic acid affects consumer acceptability after extended storage rather than immediately post-production.

#### 4.1.2. Data Gaps and Availability

Despite identifying a large number of surveys (*n* = 71) with potential data, there were very few datasets openly accessible for analysis or containing reports with information necessary to describe the proportion of the population consuming particular foods. In the end, only 22% (18/81) of LILMICs had data to identify the potential coverage of alternative food vehicles; if all countries with potentially relevant surveys (including those in Supplementary Table S7, with unverified surveys) had available datasets for analysis or eligible reports, 75% (61/81) of LILMICs would have information on food coverage. All available datasets came from the World Bank Microdata Library, International Household Survey Network, or national statistics sites that seemed to run on platforms such as the World Bank Microdata Library. On the other hand, only one dataset came from a national consumption or nutrition survey. The available datasets also did not represent the most recent surveys conducted in the country. This lack of data transparency and access severely limits the ability of global development efforts to use data to drive decision-making across a multitude of sectors. In this case, it limited the ability to identify foods that may have greater coverage than the existing three cereal grains and potentially tailor a national food fortification program to a country’s dietary patterns. Current efforts to expand access to dietary databases, such as the Global Dietary Database and the WHO/FAO Global Individual Food Consumption Tool, could provide additional data to identify potential alternative food vehicles.

#### 4.1.3. Coverage Is Only One Piece of Identifying a Food Vehicle’s Opportunity for Fortification

High coverage across a population is an important criterion to determine whether a food is a good vehicle for reaching most of the population. However, a food’s true opportunity depends on its feasibility for fortification—in particular, whether it is industrially produced, and its coverage among and the amount consumed by vulnerable populations. Our work provides a sense of which foods and in which countries it may be valuable to conduct an industry landscape analysis to inform feasibility [78], filling research gaps to fortifying novel foods with folic acid, and on a global basis, how to potentially prioritize novel foods for these activities.

### 4.2. Objective 2: Systematic Review of Research on Fortifying Alternative Foods with Folic Acid

#### Food Processing Considerations in Evaluating Evidence Gaps

Understanding a food industry’s landscape (e.g., whether milk is industrially produced) is a first step in describing the feasibility of fortifying a food from an implementation standpoint. However, understanding a nutrient’s potential viability in a food vehicle requires understanding the food’s production process within that industry and a population’s storage, preparation, and dietary practices with that food.

Fortifying food within a centralized, industrial food processing system is more efficient than in a system with many small-scale producers or processors [79]. Relying on a centralized process allows for many advantages, including greater economies of scale in fortification expenses (e.g., purchasing of vitamin and mineral premix and fortification equipment, ability to invest in more modern processes such as automation), widespread distribution (broad food access by consumers), and more efficient regulatory monitoring (smaller number of manufacturing facilities to inspect by the government).

However, industrially processed foods may also have more complicated supply chains, in which a fortified food may go through many steps before it reaches the consumer. For example, supply chains to produce a food’s derivative product could be more varied and fragmented than a base food (e.g., more biscuit producers than wheat flour millers in a country; in the case of yogurt and milk, this may be the opposite in some contexts—many milk producers and a consolidated yogurt-producing industry). Fortifying a food at the most consolidated point of food processing facilitates regulatory monitoring—thus, it is worthwhile to assess a product’s supply chain process to pinpoint at what stage of food production fortification should occur and the associated research gaps to understand a food’s feasibility for fortification with folic acid. Based on experiences with other staple foods fortified with folic acid, food fortification stakeholders (particularly the food industry) would like to see evidence that fortifying a food will not result in significant/unacceptable changes to production processes and food characteristics [80] and that the investments and resources put into fortifying foods will confidently be translated into benefits to the public’s health (and attributable to the fortified food) [81]. As such, when evaluating any novel food–nutrient combination as an opportunity for fortification, it is essential to consider the breadth of the evidence available along a product’s production and supply chain and whether there are substantial gaps in evidence. The variety of foods studied demonstrates the diversity in food processing that folic acid must potentially undergo in novel foods and flags certain circumstances in which folic acid losses were elevated (e.g., acidity, presence of ethanol).

Furthermore, although a food may have been studied for several relevant outcomes, if the breadth of research is limited to one research group or the results of one Ph.D. dissertation, it may be worthwhile to look more closely at methodologies used to ensure that no issues regarding the validity/reproducibility of results are raised.

To identify where these research needs may be, we need to consider that there are simplistically three main stages in a food’s lifecycle, which (with the exception of consumption) may occur multiple times throughout that cycle: production and/or processing/preparation, storage, and consumption. Multiple outcomes may be relevant at each stage, depending on that food’s lifecycle. For example, if a food is typically stored for an extended period before further processing or after reaching the commercial market, it may be important to assess nutrient retention and any potential impact that storage may have on food characteristics or acceptability (e.g., whether a nutrient is found to interact with these two aspects over a period of storage—this was not found to be the case for folic acid in most of the literature assessing storage impacts on food characteristics or acceptability). The same can be said for any stage in food processing or preparation: if a fortified food is an ingredient in a commonly consumed processed food that undergoes intensive food processing (e.g., extrusion, high-heat treatment), food processors may want to understand nutrient retention and/or food characteristics or acceptability. The final research needs in a novel food-nutrient combination are understanding whether the food is biologically available (efficacious) and if consumers find it acceptable (similar or preferred) compared to the non-fortified version of that food.

Notably, although we have so far discussed potential alternative vehicles as though they are homogenous food categories, there may be differing effects of fortification on nutrient retention, food characteristics, or acceptability within a food vehicle’s subtypes (e.g., canola oil vs. palm oil; coarse salt vs. refined salt) if these subtypes have significantly different uses in food preparation or desired characteristics from consumers. If multiple subtypes of a food vehicle are widely used in a country or population, then exploring the technological feasibility of food vehicle subtypes is also recommended.

However, there should be a balance when filling research needs to answer the technological feasibility of any given novel food-nutrient combination; it is not reasonable to expect studies conducted along every single step in a food’s lifecycle, and evidence gaps should not be justification alone to avoid moving forward with a novel food–nutrient combination. There are 15 nutrients specified in fortification standards across oil, maize flour, rice, salt, and wheat flour [13]. Reasonable evidence and experiences fortifying other foods with the same nutrients should be considered before placing an impractical burden of proof on a novel food–nutrient combination. In the case of folic acid in maize flour, rice, and wheat flour, despite the difference in supply chains across many countries for rice, flour, and foods made with flour, there has been a consistent reduction in NTDs found in countries after the fortification of flour with folic acid [82].

### 4.3. Study Limitations

This work was limited to food or human outcomes directly related to fortifying the food with folic acid. However, in current global program practice, folic acid is never the sole nutrient added to a food vehicle [13]. In the case of multi-nutrient fortified food vehicles, although there are no known concerns regarding nutrient-nutrient interactions between folic acid and other nutrients in maize flour, rice, or wheat flour, in the case of novel methods or novel foods, there may be relevant considerations. Multi-nutrient fortified foods were included in the current research in non-efficacy outcomes; in some of these cases, researchers reported high nutrient losses for iodine and vitamin A when co-fortified with folic acid in salt and sugar, respectively [48]; on the other hand, there were minimal or no losses in salt co-fortified with iodine and folic acid [47,50]. Although folic acid is often co-fortified with other nutrients in cereal grains, it may be a concern raised in different foods, particularly where new technologies to fortify the food are required (e.g., vacuum impregnation for fruit fortification [63,64,65]).

Our designation of a ‘potential alternative vehicle’ may be considered limited; this is a conservative definition that only considered foods without existing mandatory fortification with folic acid, and the food was any cereal grain, oil, sugar, condiment, or milk. Our systematic review found novel methods to fortify other foods with folic acid (e.g., fortified chicken feed to increase folate content in eggs, vacuum impregnation for fruit fortification), and there are other novel foods which may be worthwhile to explore further, given their high coverage (e.g., tea, coffee).

## 5. Conclusions

Data transparency and availability to describe the coverage of alternative food vehicles are poor for LILMICs. There is evidence that many countries have data available through existing national nutrition surveys or household income and expenditure surveys. Still, their reports often do not present their results in a way that could inform fortification programs (e.g., coverage of foods that potentially could be fortifiable, disaggregated by subnational populations where possible), and datasets for re-analysis were not available in the majority of surveys identified.

Where data were available, we found that foods with the highest population coverage were oil (33.2–95.7%), sugar (32.2–98.4%), salt (49.8–99.9%), rice (32.9–97.3%), wheat flour/products (30.7–68.3%), and milk (31–78%). Excluding rice and wheat flour as novel foods, there is no (oil) or limited (salt, sugar, milk) research on fortifying these foods with folic acid. As such, in countries where cereal grains are not an opportunity for large-scale food fortification, a key barrier to moving forward with fortifying alternative, high coverage, staple food vehicles with folic acid is a lack of understanding regarding efficacy, nutrient retention, and potential effects on the food’s characteristics and acceptability (as well as foods prepared with the fortified ingredient).

## Figures and Tables

**Table 1:** 18 countries/surveys with consumption or expenditure data reported or analyzed.

Country	Survey Name, Year	Type of Data *	Survey Definition of Apparent Consumption	Food (Mandatory/Voluntary, Year ^†^) [9]
Bolivia [17]	Household Survey 2017 *(Encuesta de Hogares)*	AC/E	Past mo	Wheat flour (M, 1996)
Burundi [18]	Burundi National Nutrition Survey 2005 *(Rapport de l’Enquête Nationale de Nutrition de la Population)*	AC	Past 7 d	Wheat flour (M, 2015) Maize flour (M, 2015)
El Salvador [19]	Multipurpose Household Survey 2014 *(Encuesta de Hogares de Propósitos Múltiples)*	E	Past mo	Wheat flour (M, 2017) Maize flour (M, 2013)
Ethiopia [20]	Socioeconomic Survey 2015–2016, Wave 3	AC	Past 7 d	Wheat flour (V, 2017)
Gambia, The [21]	Integrated Household Survey 2015	AC ^‡^	Past 7 d	N/A
India [22]	National Sample Survey 68th round, Household Consumption of Various Goods and Services in India 2011–2012	AC	Past 30 d/Past 7 d	Wheat flour (V, 2016)
Kosovo [23]	Living Standards Measurement Survey 2000	E	Past mo	Wheat flour (M, 2012)
Liberia [24]	Household Income and Expenditure Survey 2016	AC	Past 7 d	Wheat flour (M, 2017)
Malawi [25]	Fourth Integrated Household Survey 2016–2017	AC	Past 7 d	Wheat flour (M, 2015) Maize flour (M, 2015)
Mozambique [26]	Household Budget Survey 2008–2009 *(Inquérito aos Agregados Familiares)*	AC	Past 7 d	Wheat flour (M, 2016) Maize flour (M, 2016)
Niger [27]	National Survey on Household Living Conditions and Agriculture 2014, Wave 2 Panel Data *(l’Enquête Nationale sur les Conditions de Vie des Ménages et l’Agriculture)*	“Use”	Past 7 d	Wheat flour (M, 2010)
Nigeria [28]	Nigeria—General Household Survey, Panel 2015–2016, Wave 3	AC ^‡^	Past 7 d	Wheat flour (M, 2010) Maize flour (M, 2010)
Philippines ^§^ [16]	National Nutrition Survey 2008	C	2 d, non-consecutive 24 h recall; adults aged 20–59 y	N/A
Sierra Leone [29]	Integrated Household Survey 2011	AC	Past y	Wheat flour (V, 2010)
Somaliland [30]	Somaliland Household Survey 2013, Adapted for the Somali High Frequency Survey	AC	Past 7 d	N/A
Tajikistan [31]	Living Standards Survey 2009	AC	Past 7 d	N/A
Tanzania [32]	National Panel Survey 2014–2015, Wave 4	AC ^‡^	Past 7 d	Wheat flour (M, 2011) Maize flour (M, 1975)
Uganda [33]	Uganda Living Standards Measurement Survey—Integrated Survey on Agriculture 2013–2014 (National Panel Survey 2013/14)	AC	Past 7 d	Wheat flour (M, 2005) Maize flour (M, 2011)

N/A, not applicable (neither mandatory nor voluntary fortification of any foods with folic acid). * Type of data: AC, apparent consumption if consumption is collected through an income and expenditure survey; C, if consumption is collected using a dietary intake assessment tool; E, expenditure; if an income and expenditure survey refers to both purchase and consumption, then AC/E is listed (e.g., “In the last month in your home did you buy, get or consume (…)?”). For Niger, the questionnaire neither uses consume nor purchase, but “use”. ^†^ The Global Fortification Data Exchange (GFDx) tracks the year that mandatory fortification was passed. However, food standards containing fortification requirements are usually separate legal instruments that may be updated on a much more frequent basis. For this reason, GFDx does not track the historical changes in fortification standards. For foods with mandatory fortification legislation, the years refer to the year of the legislation; it is possible that the inclusion of folic acid in a standard may have been implemented at a later date. For foods under voluntary fortification, the year refers to the most recent standard available. ^‡^ Excluded foods consumed outside of the home. ^§^ In all countries except for the Philippines, all consumption or expenditure, data were at the household level rather than that of individuals. In the Philippines, pregnant women and lactating women were described separately, but not women of reproductive age [16].

**Table 2:** Potential alternative food vehicles for folic acid with coverage of ≥ 30% by frequency and by countries *.

Food	Occurrence	Countries
Oil	16	Bolivia, Burundi, El Salvador, Ethiopia, Gambia, India, Liberia, Malawi, Mozambique, Niger, Nigeria, Philippines, Sierra Leone, Somaliland, Tajikistan, Tanzania
Sugar	16	Bolivia, Burundi, El Salvador, Ethiopia, Gambia, India, Liberia, Malawi, Mozambique, Niger, Nigeria, Philippines, Sierra Leone, Somaliland, Tajikistan, Tanzania
Salt	14	Bolivia, El Salvador, Ethiopia, Gambia, India, Liberia, Malawi, Mozambique, Niger, Nigeria, Sierra Leone, Somaliland, Tajikistan, Tanzania
Rice	12	Bolivia, El Salvador, Gambia, India, Liberia, Niger, Nigeria, Philippines, Sierra Leone, Somaliland, Tajikistan, Tanzania
Wheat flour ^†^	7	Gambia, India, Kosovo, Philippines, Sierra Leone, Somaliland, Tajikistan
Milk (liquid, powder, or unspecified)	6	Bolivia, Ethiopia, India, Kosovo, Somaliland, Tajikistan
Bouillon cubes	3	Gambia, Liberia, Niger
Maize flour	3	Ethiopia, Kosovo, Niger
Millet	3	Gambia, Niger, Somaliland
Sorghum	2	Ethiopia, Nigeria
Teff	1	Ethiopia

* Refers to the countries with identified surveys in Table 1. ^†^ Includes countries with voluntary fortification standards for wheat flour that include folic acid.

**Table 3:** Studies fortifying novel food with folic acid by food category, specific food, outcomes studied, and whether other nutrients were added * (*n* = 34).

Reference	Food Studied	Study Objective/Design	Fortified with MMN?
**Dairy products (*n* = 10)**			
Kelly et al., 1997 [35]	Low-fat milk	Efficacy (no control/placebo), single meal	No
Achanta et al., 2007 [36]	Low-fat milk	Acceptability, food characteristics, and sensory changes after storage	No
Keane et al., 1998 [37]	Milk	Efficacy (controlled, six-month feeding)	No
Green et al., 2005 [38]	Milk powder	Efficacy (controlled, three-month feeding)	No
de Jong et al., 2005 [39]	Milk (pasteurized and UHT)	Efficacy (controlled, one-month feeding)	No
Aryana, 2003 [40]	Yogurt (fat-free, sugar-free, unflavored)	Food characteristics and sensory changes after storage	No
Boeneke et al., 2007 [41]	Yogurt (fat-free, lemon-flavored)	Food characteristics and sensory changes after storage	No
Pérez-Esteve et al., 2016 [42]	Yogurt (stirred ^†^)	Food characteristics and sensory changes after storage	No
Gaur et al., 2018 [43]	Traditional Indian yogurt-based drink (*chhansh*)	Food characteristics and sensory changes after storage	Yes
de Jong et al., 2000 [34]	Vanilla custard, vanilla–mixed fruit quark, strawberry yogurt, vanilla–apple yogurt	Folic acid retention after storage	Yes
**Condiments, spreads (*n* = 7)**			
Pentieva et al., 2003 [44]	Low-fat spread	Efficacy (cross-over, one-week feeding)	No
Vinodkumar et al., 2009b [45]	Salt	Folic acid retention after storage and cooking	No
Vinodkumar et al., 2009a [46]	Salt	Folic acid retention after storage	Yes
Sangakkara, 2011 [47]	Salt	Folic acid retention after production	Yes
Li et al., 2011 [48]	Salt and sugar	Folic acid retention after storage; product color stability after storage	Yes
McGee, 2012 [49]	Salt	Food characteristics after fortification, folic acid retention after production	Yes
McGee et al., 2017 [50]	Salt	Food characteristics after fortification, folic acid retention in production	Yes
**Beverages/fruit products (*n* = 7)**			
Öhrvik et al., 2008 [51]	Orange juice	Folic acid retention after storage	Yes
Rivas et al., 2007 [52]	Orange juice and milk (mixed)	Folic acid retention after storage	Yes
Ruiz-Rico et al., 2017 [53]	Apple and orange juice	Folic acid retention after storage	No
Frommherz et al., 2014 [54]	Commonly sold vitamin juices	Folic acid retention after storage	Yes
Kida et al., 2018 [55]	Acidic beverage	Folic acid retention after storage	No
N. de Jong et al., 2000 [34]	Orange–peach juice, apple–berry-grape juice, apple compote, apple–peach compote	Folic acid retention after storage	Yes
Tapola et al., 2004 [56]	Mineral water	Folic acid retention after storage	Yes
**Meat and eggs (*n* = 6)**			
House et al., 2002 [57]	Chicken eggs	Folic acid retention after storage	No
Altic et al., 2016 [58]	Chicken eggs	Folic acid retention after storage and cooking	No
Galán et al., 2010 [59]	Hamburgers (fresh beef meat)	Acceptability and food and sensory characteristics after fortification	No
Galán et al., 2011b [60]	Cooked sausages (*mortadella*)	Folic acid retention during and after production; acceptability and food and sensory characteristics after fortification	No
Galán et al., 2011a [61]	Fermented dry ready-to-eat sausages (*salchichón*)	Folic acid retention during and after production; acceptability and food and sensory characteristics after fortification	No
Galán et al., 2013 [62]	Fresh hamburger, cooked sausages, and dry fermented sausages	Folic acid retention after storage	No
**Fruit (*n* = 3)**			
Peña Correa et al., 2013 [63]	Cape gooseberry (fresh)	Food characteristics after fortification	Yes
Tapia et al., 2014 [64]	Papaya slices (fresh)	Acceptability and food characteristics after fortification	Yes
Moreno et al., 2016 [65]	Apple slices (dried)	Food characteristics after fortification	No
**Cereal grains (*n* = 1)**			
Tripathi et al., 2011 [66]	Finger millet flour	Food characteristics after storage	Yes
**Candy (*n* = 1)**			
Pallavi et al., 2014 [67]	Indian traditional sweet (peanut *chikki*)	Food characteristics after fortification	Yes

MMN, Multiple micronutrients (in addition to folic acid); UHT, ultra-high temperature processing. * Novel defined as foods other than maize flour, rice, and wheat flour (and their derivatives). Studies within categories are ordered by food type and publication year. ^†^ Stirring, as defined by authors: where the yogurt’s “structure is disintegrated by shearing processes before packing, facilitates the incorporation of new ingredients such as fruits, fibers and [sic] others relevant compounds”.

## Data Availability

The datasets analyzed during this study are both publicly and privately available household expenditure/consumption survey datasets. If publicly available, the datasets were downloaded or linked to from the World Bank’s Microdata Library (https://microdata.worldbank.org/index.php/home) or the country’s own microdata catalog. The World Bank Microdata Library also provides contact information if the datasets are unavailable for direct download.

## Supplementary Materials

The following supporting information can be downloaded at: https://www.mdpi.com/article/10.3390/nu16091312/s1, Tables S1–S10: Percent of households reported consuming foods in dietary surveys or household expenditure and consumption surveys, Supplemental document: Protocol.

## Abbreviations

GHDx: Global Health Data Exchange; LILMICs: Low income, and lower-middle-income countries; NTD: neural tube defect.

## References

[1] Allen L.H., De Benoist B., Dary O., Hurrell R.F., World Health Organization, Food and Agriculture Organization of the United Nations (2006). Guidelines on Food Fortification with Micronutrients.

[2] Bürgi H., Supersaxo Z., Selz B. (1990). Iodine deficiency diseases in Switzerland one hundred years after Theodor Kocher’s survey: A historical review with some new goitre prevalence data. Acta Endocrinol..

[3] National Academies Press (2003). Dietary Reference Intakes: Guiding Principles for Nutrition Labeling and Fortification.

[4] Czeizel A.E., Dudás I. (1992). Prevention of the First Occurrence of Neural-Tube Defects by Periconceptional Vitamin Supplementation. N. Engl. J. Med..

[5] (1991). Prevention of neural tube defects: Results of the Medical Research Council Vitamin Study. MRC Vitamin Study Research Group. Lancet.

[6] Berry R.J., Li Z., Erickson J.D., Li S., Moore C.A., Wang H., Mulinare J., Zhao P., Wong L.-Y.C., Gindler J. (1999). Prevention of Neural-Tube Defects with Folic Acid in China. N. Engl. J. Med..

[7] Alasfoor D., Elsayed M.K., Mohammed A.J. (2010). Spina bifida and birth outcome before and after fortification of flour with iron and folic acid in Oman. East. Mediterr. Health J..

[8] Food and Drug Administration Food (1996). Food and Drug Administration Food Standards: Amendment of Standards of Identity for Enriched Grain Products to Require Addition of Folic Acid.

[9] Global Fortification Data Exchange Fortification Legislation. https://fortificationdata.org/interactive-map-fortification-legislation/#.

[10] Castillo-Lancellotti C., Tur J.A., Uauy R. (2013). Impact of folic acid fortification of flour on neural tube defects: A systematic review. Public. Health Nutr..

[11] Food and Agriculture Organization What Do People Eat?. http://www.fao.org/3/u8480e/u8480e07.htm.

[12] Tessema M., Moges T., Zerfu D., W/Yohannes M., Mulugeta A., Belachew T., Hadis M. (2019). Preventing Neural Tube Defects in Ethiopia: An Issue Brief.

[13] Global Fortification Data Exchange Count of Nutrients in Fortification Standards. http://www.fortificationdata.org.

[14] Veritas Health Innovation (2024). Covidence Systematic Review Software.

[15] Smithells R.W., Seller M.J., Harris R., Fielding D.W., Schorah C.J., Nevin N.C., Sheppard S., Read A.P., Walker S., Wild J. (1983). Further experience of vitamin supplementation for prevention of neural tube defect recurrences. Lancet.

[16] Philippines Food and Nutrition Research Institute (2008). 7th National Nutrition Survey: 2008.

[17] Estado Plurinacional de Bolivia, Instituto Nacional de Estadística Encuesta de Hogares 2017 Catálogo ANDA: Catálogo Central de Datos y Microdatos. http://anda.ine.gob.bo/index.php/catalog/55.

[18] Ministere de la Sante Publique, Republic du Burundi, World Food Programme, Food and Agriculture Organization, US Agency for International Development, UNICEF (2005). Rapport de L’enquête Nationale de Nutrition de la Population.

[19] Ministerio de Economía, Gobierno de El Salvador, Dirección General de Estadística y Censos (2014). Encuesta de Hogares de Propósitos Múltiples 2014.

[20] Central Statistical Agency of Ethiopia Socioeconomic Survey 2015–2016, Wave 3: The World Bank Microdata Library. https://microdata.worldbank.org/index.php/catalog/2783.

[21] The Gambia Bureau of Statistics Integrated Household Survey 2015: The World Bank Microdata Library. https://datacatalog.worldbank.org/dataset/gambia-integrated-household-survey-2015/resource/1ab5933c-b8d8-4b0d-8a32-8fe03424a25a.

[22] Ministry of Statistics and Programme Implementation (2014). National Sample Survey 68th round, Household Consumption of Various Goods and Services in India 2011–2012.

[23] The World Bank Kosovo Living Standards Measurement Survey 2000: The World Bank Microdata Library. https://microdata.worldbank.org/index.php/catalog/77.

[24] Liberia Institute for Statistics and Geo-Information Services Household Income and Expenditure Survey 2016: The World Bank Microdata Library. http://microdata.worldbank.org/index.php/catalog/2986.

[25] Malawi National Statistical Office Fourth Integrated Household Survey 2016–2017: The World Bank Microdata Library. https://microdata.worldbank.org/index.php/catalog/2936/study-description.

[26] Mozambique Direcção de Censos e Inquéritos Inquérito sobre Orçamento Familiar 2008–2009: International Household Survey Network. http://catalog.ihsn.org/index.php/catalog/2168/study-description#page=accesspolicy&tab=study-desc.

[27] Niger Survey and Census Division National Survey on Household Living Conditions and Agriculture 2014: The World Bank Microdata Library. https://microdata.worldbank.org/index.php/catalog/2676.

[28] Nigeria National Bureau of Statistics General Household Survey, Panel 2015–2016, Wave 3: World Bank Microdata Library. http://microdata.worldbank.org/index.php/catalog/2734.

[29] Statistics Sierra Leone Integrated Household Survey 2011: The World Bank Microdata Library. https://microdata.worldbank.org/index.php/catalog/2943.

[30] The World Bank Somaliland Household Survey 2013, Adapted for the Somali High Frequency Survey: The World Bank Microdata Library. https://microdata.worldbank.org/index.php/catalog/2818.

[31] Tajikistan State Statistical Agency Living Standards Survey 2009: The World Bank Microdata Library. http://microdata.worldbank.org/index.php/catalog/73.

[32] Tanzania National Bureau of Statistics National Panel Survey 2014–2015, Wave 4: The World Bank Microdata Library. https://microdata.worldbank.org/index.php/catalog/2862.

[33] Uganda Bureau of Statistics National Panel Survey 2013–2014: The World Bank Microdata Library. https://microdata.worldbank.org/index.php/catalog/2663.

[34] De Jong R.J., Susan G.M., Adam L.C.P.G.M., De Groot C.D.G., Gerrit J Hiddink M.S.A., Van Daatselaar N. (2000). Variability of micronutrient content in enriched dairy and fruit products. Int. J. Food Sci. Nutr..

[35] Kelly P., McPartlin J., Goggins M., Weir D.G., Scott J.M. (1997). Unmetabolized folic acid in serum: Acute studies in subjects consuming fortified food and supplements. Am. J. Clin. Nutr..

[36] Achanta K., Boeneke C.A., Aryana K.J. (2007). Characteristics of Reduced Fat Milks As Influenced By the Incorporation of Folic Acid. J. Dairy. Sci..

[37] Keane E.M., O’Broin S., Kelleher B., Coakley D., Walsh J.B. (1998). Use of Folic Acid-Fortified Milk in the Elderly Population. Gerontology.

[38] Green T.J., Skeaff C.M., Rockell J.E.P., Venn B.J. (2005). Folic acid fortified milk increases blood folate and lowers homocysteine concentration in women of childbearing age. Asia Pac. J. Clin. Nutr..

[39] De Jong N., Verwei M., West C.E., van Vliet T., Siebelink E., van den Berg H., Castenmiller J.J.M. (2005). Bioavailability of folic acid from fortified pasteurised and UHT-treated milk in humans. Eur. J. Clin. Nutr..

[40] Aryana K.J. (2003). Folic acid fortified fat-free plain set yoghurt. Int. J. Dairy. Tech..

[41] Boeneke C.A., Aryana K.J. (2008). Effect of folic acid fortification on the characteristics of lemon yogurt. LWT Food Sci. Technol..

[42] Pérez-Esteve É., Ruiz-Rico M., Fuentes A., Marcos M.D., Sancenón F., Martínez-Máñez R., Barat J.M. (2016). Enrichment of stirred yogurts with folic acid encapsulated in pH-responsive mesoporous silica particles: Bioaccessibility modulation and physico-chemical characterization. LWT Food Sci. Technol..

[43] Gaur S., Waller A., Andrade J. (2018). Effect of Multiple Micronutrient Fortification on Physico-Chemical and Sensory Properties of Chhash (Traditional Indian Yogurt-Based Drink). Foods.

[44] Pentieva K., McKillop D., Duffy N., de Deckere E.A.M., Jacobs R.G.J.M., van der Put N.M.J., McNulty H. (2003). Acute absorption of folic acid from a fortified low-fat spread. Eur. J. Clin. Nutr..

[45] Vinodkumar M., Rajagopalan S. (2009). Multiple micronutrient fortification of salt. Eur. J. Clin. Nutr..

[46] Vinodkumar M., Erhardt J.G. (2009). Rajagopalan Impact of a Multiple-micronutrient Fortified Salt on the Nutritional Status and Memory of Schoolchildren. Int. J. Vitam. Nutr. Res..

[47] Sangakkara A.R. (2011). Double Fortification of Salt with Folic Acid and Iodine.

[48] Li Y.O., Diosady L.L., Wesley A.S. (2011). Folic Acid Fortification through Existing Fortified Foods: Iodized Salt and Vitamin A—Fortified Sugar. Food Nutr. Bull..

[49] McGee J.T. (2012). The Fortification of Salt with Iodine, Iron, and Folic Acid.

[50] McGee E.J.T., Sangakkara A.R., Diosady L.L. (2017). Double fortification of salt with folic acid and iodine. J. Food Eng..

[51] Öhrvik V., Witthöft C. (2008). Orange juice is a good folate source in respect to folate content and stability during storage and simulated digestion. Eur. J. Nutr..

[52] Rivas A., Rodrigo D., Company B., Sampedro F., Rodrigo M. (2007). Effects of pulsed electric fields on water-soluble vitamins and ACE inhibitory peptides added to a mixed orange juice and milk beverage. Food Chem..

[53] Ruiz-Rico M., Pérez-Esteve É., Lerma-García M.J., Marcos M.D., Martínez-Máñez R., Barat J.M. (2017). Protection of folic acid through encapsulation in mesoporous silica particles included in fruit juices. Food Chem..

[54] Frommherz L., Martiniak Y., Heuer T., Roth A., Kulling S.E., Hoffmann I. (2014). Degradation of folic acid in fortified vitamin juices during long term storage. Food Chem..

[55] Kida K., Tomotake M., Sasako H., Matsuda Y., Sasaki N., Yamamoto N. (2018). Small amounts of ethanol attenuate folic acid stability in acidic beverages during storage. Food Sci. Nutr..

[56] Tapola N.S., Karvonen H.M., Niskanen L.K., Sarkkinen E.S. (2004). Mineral water fortified with folic acid, vitamins B6, B12, D and calcium improves folate status and decreases plasma homocysteine concentration in men and women. Eur. J. Clin. Nutr..

[57] House J., Braun K., Ballance D., O’Connor C., Guenter W. (2002). The enrichment of eggs with folic acid through supplementation of the laying hen diet. Poult. Sci..

[58] Altic L., McNulty H., Hoey L., McAnena L., Pentieva K. (2016). Validation of Folate-Enriched Eggs as a Functional Food for Improving Folate Intake in Consumers. Nutrients.

[59] Galán I., García M.L., Selgas M.D. (2010). Effects of irradiation on hamburgers enriched with folic acid. Meat Sci..

[60] Galán I., García M.A.L., Selgas M.D. (2011). Irradiation is useful for manufacturing ready-to-eat cooked meat products enriched with folic acid. Meat Sci..

[61] Galán I., García M.L., Selgas M.D. (2011). Effects of ionising irradiation on quality and sensory attributes of ready-to-eat dry fermented sausages enriched with folic acid: Folic acid in ready-to-eat irradiated sausages. Int. J. Food Sci. Technol..

[62] Galán I., García M.L., Selgas M.D. (2013). Effects of the storage time on the folic acid added to ready-to-eat meat products manufactured by irradiation. Radiat. Phys. Chem..

[63] Peña Correa R.F., Cortés Rodriguez M., Montoya C.O.I. (2013). Evaluation of the physicochemical, physical, and sensory properties of fresh cape gooseberry and vacuum impregnated with physiologically active components. Vitae Rev. Fac. Química Farm..

[64] Tapia M.S., Olaizola C., Carmona A., Martín-Belloso O., Reynes M. (2014). A novel functional fresh-cut product of papaya (carica papaya l. ’Maradol’) using vacuum impregnation and edible coatings. Acta Hortic..

[65] Moreno J., Espinoza C., Simpson R., Petzold G., Nuñez H., Gianelli M.P. (2016). Application of ohmic heating/vacuum impregnation treatments and air drying to develop an apple snack enriched in folic acid. Innov. Food Sci. Emerg. Technol..

[66] Tripathi B., Platel K. (2011). Iron fortification of finger millet (Eleucine coracana) flour with EDTA and folic acid as co-fortificants. Food Chem..

[67] Pallavi B.V., Chetana R., Reddy S.Y. (2014). Processing, physico-chemical, sensory and nutritional evaluation of protein, mineral and vitamin enriched peanut chikki—An Indian traditional sweet. J. Food Sci. Technol..

[68] Centeno Tablante E., Pachón H., Guetterman H.M., Finkelstein J.L. (2019). Fortification of wheat and maize flour with folic acid for population health outcomes. Cochrane Database Syst. Rev..

[69] De Paiva Azevedo E.P., dos Santos Alves E.M., Khan S.D.S., Silva L.D.S.S., de Souza J.R.B., Santos B.S., Rabelo C.B.-V., dos Santos Costa A.C., de Azevedo Filho C.A., da Silva Vasconcelos M.A. (2020). Folic acid retention evaluation in preparations with wheat flour and corn submitted to different cooking methods by HPLC/DAD. PLoS ONE.

[70] Hemery Y.M., Fontan L., Laillou A., Jallier V., Moench-Pfanner R., Avallone S., Berger J. (2020). Influence of storage conditions and packaging of fortified wheat flour on microbial load and stability of folate and vitamin B12. Food Chem. X.

[71] Hackl L.S., Abizari A.R., Speich C., Zungbey-Garti H., Cercamondi C.I., Zeder C., Zimmermann M.B., Moretti D. (2019). Micronutrient-fortified rice can be a significant source of dietary bioavailable iron in schoolchildren from rural Ghana. Sci. Adv..

[72] Pietrzik K., Bailey L., Shane B. (2010). Folic Acid and L-5-Methyltetrahydrofolate: Comparison of Clinical Pharmacokinetics and Pharmacodynamics. Clin. Pharmacokinet..

[73] Wadman M. (2019). Beset by neural tube defects, Ethiopia may fortify salt. Science.

[74] Kancherla V., Tsang B., Wagh K., Dixon M., Oakley G.P. (2020). Modeling shows high potential of folic acid-fortified salt to accelerate global prevention of major neural tube defects. Birth Defects Res..

[75] Fiedler J.L., Smitz M.-F., Dupriez O., Friedman J. (2008). Household Income and Expenditure Surveys: A Tool for Accelerating the Development of Evidence-Based Fortification Programs. Food Nutr. Bull..

[76] Weisell R., Dop M.C. (2012). The Adult Male Equivalent Concept and its Application to Household Consumption and Expenditures Surveys (HCES). Food Nutr. Bull..

[77] Zezza A., Carletto C., Fiedler J.L., Gennari P., Jolliffe D. (2017). Food counts. Measuring food consumption and expenditures in household consumption and expenditure surveys (HCES). Introduction to the special issue. Food Policy.

[78] Folate Task Team (2019). Supply Chain Analyses to Assess the Feasibility of National Food Fortification Programs: Knowledge Brief.

[79] Mkambula P., Mbuya M.N.N., Rowe L.A., Sablah M., Friesen V.M., Chadha M., Osei A.K., Ringholz C., Vasta F.C., Gorstein J. (2020). The Unfinished Agenda for Food Fortification in Low- and Middle-Income Countries: Quantifying Progress, Gaps and Potential Opportunities. Nutrients.

[80] Darnton-Hill I. (1998). Overview: Rationale and Elements of a Successful Food-Fortification Programme. Food Nutr. Bull..

[81] Das J.K., Salam R.A., Mahmood S.B., Moin A., Kumar R., Mukhtar K., Lassi Z.S., Bhutta Z.A. (2019). Food fortification with multiple micronutrients: Impact on health outcomes in general population. Cochrane Database Syst. Rev..

[82] Keats E.C., Neufeld L.M., Garrett G.S., Mbuya M.N.N., Bhutta Z.A. (2019). Improved micronutrient status and health outcomes in low- and middle-income countries following large-scale fortification: Evidence from a systematic review and meta-analysis. Am. J. Clin. Nutr..

