# Supplementary material for: Expanding Fortification with Folic Acid: Thinking Outside the Cereal-Grain Box
Source: Nutrients. 2024 Apr 27;16(9):1312. doi: 10.3390/nu16091312 (PMC11085292; doi:10.3390/nu16091312)
Supplement: Supplementary file 1 [file nutrients-16-01312-s001.zip › Supplemental document.pdf]

**Novel food vehicles fortified with folic acid for neural tube defect prevention: a systematic review protocol**

Stadnik C, Tsang B, Pachón H
23rd May, 2019

Authors

Carlen Stadnik
Hubert Department of Global Health, Emory University
1599 Clifton Road
Atlanta, Georgia, USA 30322
carlen.stadnik@emory.edu

Becky Tsang
Food Fortification Initiative
Atlanta, GA
becky.tsang@ffinetwork.org

Helena Pachón
Food Fortification Initiative
Hubert Department of Global Health, Emory University
1599 Clifton Road
Atlanta, Georgia, USA 30322
Helena.Pachon@emory.edu

## INTRODUCTION

*Rationale:*

Due to global folic acid fortification of grains, 97 babies a day are born free of neural tube defects (NTDs). Despite this success, less than 20% of NTD cases that can be prevented through folic acid fortification are prevented. To date, foods typically fortified with folic acid are limited to the cereal grains - wheat flour, maize flour, and rice. Other foods are also commonly fortified (e.g. oil, salt) but typically not with folic acid. Identifying novel foods to fortify with folic acid would help expand the range of foods that can be fortified with folic acid and add to global efforts to prevent neural tube defects.

*Objectives:*

Reviewed published literature to identify and describe novel food vehicles—specifically, processed foods (other than cereal grains)—that can be fortified with folic acid to be passed on to individuals through dietary consumption, thereby increasing folate status (e.g., salt, sugar, soup cubes/bouillon, fish sauce, tomato paste, condiments, and others as identified).

## METHODS

### INCLUSION CRITERIA

*Eligibility criteria*: Study designs eligible by outcome. The following refers to studies that were included in this systematic review:

- **Stability/retention**: Required at least two time-points.
- **Food characteristics**:
    - Single time-point studies.
    - Consumer panel taste tests, using triangle tests, preference ratings, or other tests as defined by the authors.
    - Lab measurements of food specifications (e.g. pasting, color, ash).
- **Efficacy**:
    - Controlled feeding trials (efficacy studies) with at least two-time points (pre and post intervention).
    - Eligible control groups included: no folic acid (any other micronutrients or food properties constant). Control groups must have isolated the effect of folic acid.

*Inclusion criteria*:

- For those studies that included human participants: males and females (any age), regardless of baseline anemia or folate status.
- Folic acid supplementation through animal feed was acceptable if the purpose was to improve human health; must have included human outcomes.
- <u>Cutoffs for defining a novel food vehicle this was acceptable for this report:</u>
    - The food must be a food that already exists; not explicitly described as the creation of a new food.
    - The food did not have to be explicitly described. The description may have been vague.
    - The food did not have be fortified with folic acid alone or in combination with other micronutrients.
    - Beverages were acceptable.

    - Separate foods could have been combined to form a food, which may or may not be regularly consumed.
- Time limitation – Any research during or after 1980—around the time when folic acid research was initiated, as justified by Smithell et al. (1983).

EXCLUSION CRITERIA

- Animal studies—unless the study involved animal feeding to improve the folic acid content of a novel staple food, and demonstrated evidence of improving human health.
- Studies that involved the following:
    - Only wheat, maize, or rice fortification (i.e. no 'novel' food vehicles for folic acid; other grains may be acceptable).
    - Food derivatives of wheat flour, maize flour, or rice (e.g. baked goods, such as cookies, biscuits, noodles).
    - Supplemental/complementary foods (new foods that were created expressly to serve a nutritional purpose; food aid).
    - Point-of-use fortification (e.g. folic acid added to food at home just prior to consumption).
    - Folate production by bacteria.
    - Bio-fortification.
- Eliminated grey literature (besides theses) by restricting search to particular document types in databases when this is possible, such as Web of Science.
- Limited search to articles in the English language only.
- Studies that did not include one of the three study designs mentioned above, such as *in vitro* bio accessibility models that determined folate concentration.

Search strategy

Information sources: Systematic review of Agricultural & Environmental Science Database, CAB Abstracts, EBSCO, EMBASE, Georgia State University databases (TBD), ProQuest Dissertations and Theses Global, PubMed, Scopus (SciVerse Scopus), and Web of Science. No hand-searching of references in reviews found in results.

Search string: Medical Subject Headings (MeSH) in PubMed was be used to compile a list of synonyms for potential food vehicles to construct the search string. The following search string found items on folic acid fortification of various types of foods.

> (fortify OR fortifies OR fortified OR fortifying OR fortification OR enrich OR enriches OR enriched OR enriching OR enrichment OR enriched food OR enriched foods OR fortified food OR fortified foods OR functional food OR functional foods)
>
> AND (folic acid OR folate OR vitamin B9)
>
> AND (salt OR salts OR sugar OR sugars OR fat OR fats OR oil OR oils OR butter OR butters OR margarine OR margarines OR beverage OR beverages OR water OR juice OR juices OR milk OR condiment OR condiments OR soup OR soups

OR cube OR cubes OR bouillon OR bouillons OR sauce OR sauces OR paste OR pastes OR new OR novel OR potential OR possible OR possibility OR possibilities OR suitable OR suitability OR acceptable OR acceptability)

Risk of bias was not assessed.

Ethics review: The protocol was submitted to Emory's IRB for determination if an ethics review of the project needed to be completed.

Data extraction and management

- Data was extracted independently in duplicate from studies using a digital extraction form piloted and designed for this review.
- Data from the following domains was collected:
  - Methods: Study design, unit of randomization, participant selection, method of allocation, sample size, assessment methods for each outcome.
  - Participants: Study location, age, health status, baseline anemia and nutrient status, prior defined inclusion/exclusion criteria.
  - Intervention/exposure (as relevant for efficacy studies):
    - Folic acid dose
    - Fortification method
    - Food vehicle
    - Duration of the intervention
    - Co-interventions
    - Comparison group(s)
  - Stability/retention (as relevant for stability/retention studies):
    - Pre-post measurements
    - Retention estimates during various points in the food product's supply chain: production, storage, and cooking
    - Conditions during production and storage that may affect stability of the nutrient (e.g. light, temperature, humidity, packaging), as reported by authors.
  - Food characteristics: Taste, smell, appearance, texture, and other characteristics as described by authors.

Author roles and responsibilities

- Proposed coauthors: Carlen Stadnik (Emory University), Helena Pachon (FFI), and Becky Tsang (FFI).
- Both HP and BT supervised CS, with CS(?) as the lead author.
- CS conducted the search, removed duplicates using Endnote, and used Covidence to organize the title and abstract review.
- CS and BT reviewed titles and abstracts and full texts eligibility in duplicate; HP served as the third party adjudicator.
- CS and BT extracted data from eligible papers and reviewed each other's work.
- CS(?), BT, and HP contributed in the process of writing and reviewing of the final report and manuscript.

Outcome

The outcome of this project was a systematic review containing a table of descriptive summaries of conducted research studying potential new food vehicles for fortification with folic acid. A narrative describing each food, potential, and consideration for fortification with folic acid was also composed. This review was summarized in a full report as a deliverable for the requesting donor, the Folate Task Force, and was formatted for submission to a peer-reviewed journal.
