# Supplementary material for: Expanding Fortification with Folic Acid: Thinking Outside the Cereal-Grain Box
Source: Nutrients. 2024 Apr 27;16(9):1312. doi: 10.3390/nu16091312 (PMC11085292; doi:10.3390/nu16091312)
Supplement: Supplementary file 1 [file nutrients-16-01312-s001.zip › Supplemental Tables S1-S10.pdf]

Supplementary Table S1a: Bolivia Household Survey 2017 (*Encuesta de Hogares 2017*) (11,135 HH)*

Food	**%**	**Landscape for food****	**Comments**
Pan corriente (wheat flour roll)	89.9	WF: 80% IP	Wheat flour is mandatory
Edible oil	88.3	ND	Potential alternative vehicle
Granulated sugar	86.8	ND	Potential alternative vehicle
Potato	86.2		
Rice	86.0	ND	Potential alternative vehicle
Eggs	85.4		
Onion	84.8		
Salt	84.5	ND	Potential alternative vehicle
Tomato	84.3		
Noodle	79.5	WF: 80% IP	Wheat flour is mandatory
Chicken meat (whole, sliced)	79.3		
Banana	77.6		
Carrot	75.7		
Tea	61.4		
Liquid milk	60.3	ND	Potential alternative vehicle
Soft drink in bottle / can	59.7		
Other vegetables (squash, beans, pepper, etc.)	58.6		
Boneless beef (special cuts)	57.5		
Cheese	54.2		
Beef with bone (with fibers, second, third)	52.8		
Apple	49.2		
Cookies	46.5	WF: 80% IP	Wheat flour is mandatory
Papaya	45.9		
Natural herbs (chamomile, eucalyptus, boldo, cedrón, etc.)	44.5		
Other fruits, pineapple, lemon, mango, pear, even canned, etc.	43.4		
Other cereals (oats, cereals in flakes, etc.)	42.2		
Coffee	41.6		
Yogurt	41.1		
Chocolate-based powders (Toddy, Chocolike, etc.)	40.8		
Spices, sauces, condiments, seasonings and the like	40.0		
Lettuce	34.0		
Flour (wheat, corn, etc.)	38.3	WF: 80% IP	Wheat flour is mandatory
Ground beef (current / special)	38.3		
Quinoa	31.7		
Watermelon	31.5		
Milk powder	31.0	ND	Potential alternative vehicle

*As HH sample size was consistent across foods, the individual food sample sizes are not shown.
**The GFDx only collects data for industrially processed maize flour, oil, rice, salt, and wheat flour. Global Fortification Data Exchange. Chart: Quantity and Proportion of Industrially Processed Food Vehicles. Accessed 04/June/2019. [http://www.fortificationdata.org.]
Abbreviations: WF, wheat flour; IP, industrially processed; ND, no data

Supplementary Table S1b: Burundi National Nutrition Survey 2005 (Enquête nutritionnelle national 2005)*

Food	**%**	**Landscape for food****	**Comments**
Dark green leaves	85.7		
Beans	84.8		
Palm oil	67.1	ND	Potential alternative vehicle
Cassava	63.6		
Corn	47.0	ND	Maize flour is mandatory; Unclear if this refers to fresh corn or flour
Avocado	44.2		
Sweet potatoes (white)	40.5		
Dry fish	39.6		
Vegetable banana	35.6		

*Sample sizes for household and foods not reported
**The GFDx only collects data for industrially processed maize flour, oil, rice, salt, and wheat flour. Global Fortification Data Exchange. Chart: Quantity and Proportion of Industrially Processed Food Vehicles. Accessed 04/June/2019. [http://www.fortificationdata.org.]
Abbreviations: ND, no data

Supplementary Table S1c: El Salvador Multipurpose Household Survey 2014 *(Encuesta de Hogares de Propositos Multiples 2014)* (775,930 HH)*

Food	**%**	**Landscape for food**	**Comments**
Sugar	50.0		Potential alternative vehicle
Kitchen salt	49.8		Potential alternative vehicle
Kitchen oil	47.0		Potential alternative vehicle
Chicken eggs	45.8		
Root crops, non-starchy bulbs and mushrooms (fresh, chilled or frozen)	38.2		
Vegetables cultivated for their fruit	34.1		
Rice	32.9		Potential alternative vehicle
French bread	32.6	WF: 100% IP	Wheat flour is mandatory
Salsa, condiments	30.9		
Chicken	30.3		

*As HH sample size was consistent across foods, the individual food sample sizes are not shown.
**The GFDx only collects data for industrially processed maize flour, oil, rice, salt, and wheat flour. Global Fortification Data Exchange. Chart: Quantity and Proportion of Industrially Processed Food Vehicles. Accessed 04/June/2019. [http://www.fortificationdata.org.]
Abbreviations: WF, wheat flour; IP, industrially processed; ND, no data

Supplementary Table S1d: Ethiopia Socioeconomic Survey 2015-2016, Wave 3 (4,954 HH)

Food	**% crude**	**% adjusted**	**Landscape for food****	**Comments**
Salt	92.8	93.0	ND	Potential alternative vehicle
Red pepper (berbere)	79.7	81.9		
Oils (processed)	81.6	81.4	ND	Potential alternative vehicle
Onion	81.4	80.6		
Coffee	73.7	76.8		
Maize	52.4	54.3	ND	Potential alternative vehicle (unclear if referring to fresh corn or flour)
Teff	49.3	52.4	ND	Potential alternative vehicle
Sugar	55.1	51.2	ND	Potential alternative vehicle
Green chili pepper (kariya)	52.1	51.1		
Potato	46.9	44.2		
Horsebeans	37.1	43.6		
Greens (kale, cabbage, etc.)	44.6	43.1		
Tomato	49.7	41.2		
Wheat	40.1	40.7	WF: 55% IP	Wheat flour is voluntary
Sorghum	36.3	33.1	ND	Potential alternative vehicle (unclear if referring to grains or flour)
Milk	34.4	32.1	ND	Potential alternative vehicle
Field pea	33.1	31.2		
Tea	30.2	24.6		

*As HH sample size was consistent across foods, the individual food sample sizes are not shown.
**The GFDx only collects data for industrially processed maize flour, oil, rice, salt, and wheat flour. Global Fortification Data Exchange. Chart: Quantity and Proportion of Industrially Processed Food Vehicles. Accessed 04/June/2019. [http://www.fortificationdata.org.]
Abbreviations: WF, wheat flour; IP, industrially processed; ND, no data

Supplementary Table S1e: The Gambia Integrated Household Survey 2015

Food	**%**	**n/N**	**Landscape for food***	**Comments**
Sugar	98.4	12,781/12,984	ND	Potential alternative vehicle
Salt	93.0	11,623/12,498	ND	Potential alternative vehicle
Onion	92.0	11,923/12,962		
Maggi Cube	89.5	11,737/13,112	ND	Bouillon cube; potential alternative vehicle
Vegetable oil	85.4	11,158/13,064	ND	Potential alternative vehicle
Chinese green tea(20)(Ataya)	76.6	10,033/13,092		
Tomato puree (paste)	72.6	9,551/13,158		
Peanut butter	71.8	9,229/12,855		
Fresh Bonga (fish)	71.3	9,355/13,127		
Bread	71.2	9,331/13,115	ND	Potential alternative vehicle
Palm oil	68.7	8,989/13,092	ND	Potential alternative vehicle
Chili powder (black pepper)	52.2	6,865/13,142		
Big red pepper	51.5	6,774/13,142		
Bitter tomato	43.0	5,631/13,092		
Tea bags	43.0	5,600/13,026		
Bisap	41.9	5,188/12,397		
Smoked bonga (fish)	41.1	5,410/13,150		
Small grained rice (imported)	41.0	5,317/12,979	100% IP**	Potential alternative vehicle
Garden eggs	40.6	5,296/13,054		
Okra	40.3	5,245/13,022		
Tomatoes-fresh	39.2	5,131/13,090		
Locust beans (Neteetu)	38.1	5,013/13,152		
Small Pepper-fresh	37.2	4,870/13,109		
Small dry pepper	35.7	4,683/13,122		
Dried fish	34.9	4,581/13,138		
Millet	33.6	4,338/12,912	ND	Potential alternative vehicle
Garlic	32.2	4,242/13,157		
Biscuit	30.7	4,047/13,166	WF: 100% IP	Potential alternative vehicle

*The GFDx only collects data for industrially processed maize flour, oil, rice, salt, and wheat flour. Source: Global Fortification Data Exchange. Chart: Quantity and Proportion of Industrially Processed Food Vehicles. Accessed 04/June/2019. [http://www.fortificationdata.org.]

**Imported rice is assumed 100% industrially produced

Abbreviations: WF, wheat flour; IP, industrially processed; ND, no data

Supplementary Table S1f: National Sample Survey 68th Round, Household Consumption of Various Goods and Services in India 2011-2012 (59,678 HH)*

Food**	**%*****	**Landscape for food******	**Comments**
170 salt	98.1	ND	Potential alternative vehicle
201 onion	95.9		
254 turmeric (gm)	94.9		
200 potato	91.1		
251 garlic (gm)	89.6		
271 tea: leaf (gm)	86.1		
172 sugar – other sources	85.1	ND	Potential alternative vehicle
256 dry chilies (gm)	85.0		
102 rice – other sources	84.6	Rice: 50% IP	Voluntary fortification
207 green chilies	83.1		
252 jeera (gm)	82.2		
160 milk: liquid (litre)	78.0	ND	Potential alternative vehicle
253 dhania (gm)	74.7		
202 tomato	74.6		
250 ginger (gm)	69.4		
291 biscuits, chocolates, etc.	68.3	WF: 30% IP	Voluntary fortification (biscuits)
108 wheat/atta – other sources	67.4	WF: 30% IP	Voluntary fortification
261 other spices (gm)	64.8		
206 palak/other	61.2		
140 arhar, tur	59.6		
217 other vegetables	58.1		
203 brinjal	57.8		
181 mustard oil	52.7		
101 rice – P.D.S.	45.9	50% IP	Voluntary fortification
143 moong	45.6		
283 cooked snacks purchased	44.7		
260 oilseeds (gm)	44.6		
144 masur	41.1		
141 gram: split	39.9		
145 urd	38.9		
270 tea: cups (no.)	37.3		
220 banana (no.)	36.5		
184 refined oil	35.7	ND	Potential alternative vehicle
292 papad, bhujia, namkeen	34.9		
151 besan	34.7		
107 wheat/atta – P.D.S.	33.9	WF: 30% IP	Voluntary fortification
171 sugar – PDS	33.7	ND	Potential alternative vehicle
208 lady's finger	33.6		Assumed this is okra, not the cookie
213 gourd, pumpkin	33.2		

*As HH sample size was consistent across foods, the individual food sample sizes are not shown.
**The food's purchase/consumption reference period is 30 days for items 101-179 and 7 days for items 180-299
***% is taken from the report per 1,000 households, not the sample size estimate
**** The GFDx only collects data for industrially processed maize flour, oil, rice, salt, and wheat flour. Source: Global Fortification Data Exchange. Chart: Quantity and Proportion of Industrially Processed Food Vehicles. Accessed 04/June/2019. [http://www.fortificationdata.org.]
Abbreviations: WF, wheat flour; IP, industrially processed; ND, no data

Supplementary Table S1g: Kosovo Living Standards Measurement Survey 2000

Food	**%**	**n/N**	**Landscape for food***	**Comments**
Coffee	96.4	2,735/2,838		
Tea	94.7	2,295/2,423		
Soft drinks (Coca and juices)	92.4	2,450/2,653		
Baby formula	91.9	486/529	ND	Likely already fortified
Spices	89.9	2,101/2,337		
Yeast	87.4	2,131/2,439		
Orange and lemons	84.0	1,717/2,043		
Fast food: bureks, etc…	81.0	251/310		
Chicken	77.6	2,065/2,662		
Grapes	76.9	1,718/2,234		
Eggs	76.8	2,134/2,779		
Biscuits	75.2	1,286/1,711	WF: 70% IP	Wheat flour is mandatory
Beer	73.7	451/612		
Other fruits	67.7	598/884		
Misc. Other food expenses	66.4	279/420		
Beef/buffalo	63.7	1,320/2,073		
Bread	62.5	1,241/1,985	WF: 70% IP	Wheat flour is mandatory
Milk	62.1	1,684/2,712	ND	Potential alternative vehicle
Apples	61.1	1,405/2,300		
Pasta and rice (macaroni)	60.8	1,669/2,746	WF: 70% IP	Wheat flour is mandatory
Slivovica	59.6	274/460		
Fresh cheese	59.3	1,418/2,391		
Sweet pepper	58.0	1,618/2,791		
Cabbage	57.9	1,377/2,377		
Jam and	56.7	912/1,601		
Butter and other fat	53.4	706/1,321		
Beans	48.5	1,345/2,774		
Tomatoes	47.8	1,317/2,753		
Curd	46.2	755/1,635		
Other vegetables	45.7	301/658		
Yogurt	43.2	846/1,960		
Canned foods	41.9	242/577		
Cucumber	38.3	888/2,319		
Fish	35.0	271/774		
Garlic	33.9	737/2,173		
Pork	32.3	139/430		
Maize and other cereals (flour or grain)	30.1	339/1,127	MF: 48% IP	Potential alternative vehicle

* The GFDx only collects data for industrially processed maize flour, oil, rice, salt, and wheat flour. Source: Global Fortification Data Exchange. Chart: Quantity and Proportion of Industrially Processed Food Vehicles. Accessed 04/June/2019. [http://www.fortificationdata.org.]
Abbreviations: WF, wheat flour; IP, industrially processed; ND, no data; MF, maize flour

Supplementary Table S1h: Liberia Household Income and Expenditure Survey 2016

Food	**%**	**n/N**	**Landscape for food***	**Comments**
Salt	94.7	7,906/8,350	Salt: 100% IP	Potential alternative vehicle
Bouillon cubes (maggi, jumbo, etc)	93.9	7,842/8,350	Salt: 100% IP	Potential alternative vehicle
Palm oil	84.7	7,073/8,350	ND	Potential alternative vehicle
Onions	72.3	6,032/8,349		
Smoked fish (dried/salted)	69.7	5,819/8,350		
Palm nuts	68.1	5,686/8,350		
Bitter balls/Kitilay	67.0	5,595/8,350		
Dry pepper	64.9	5,418/8,350		
Imported Rice (including pusswa, butter rice, etc.)	62.6	5,223/8,350	100%**	Potential alternative vehicle
Potato greens/ sweet potato greens	60.9	5,087/8,349		
Cassava leaves	60.2	5,024/8,349		
Cassava roots	60.0	5,005/8,348		
Fresh pepper	59.1	4,936/8,350		
Fresh fish (cassava fish, cavalla fish, mackerel, snappers, soul fish etc.)	55.0	4,597/8,350		
Cassava flour (fufu), gari	46.2	3,856/8,349		
Local rice	42.7	3,561/8,349	Rice: 1%	Potential alternative vehicle
Chicken feet	41.7	3,483/8,350		
Okra	35.6	2,968/8,349		
Sugar	34.7	2,894/8,349	ND	Potential alternative vehicle
Argo oils/ vegetable oils / olive oil	33.2	2,768/8,349	ND	Potential alternative vehicle
Plantains	32.7	2,728/8,350		
Frozen chicken	32.3	2,695/8,350		
Wild/Bush meat (Porcupine gazelle, palm worms, chenilles, monkey meat)	31.1	2,597/8,350		

*The GFDx only collects data for industrially processed maize flour, oil, rice, salt, and wheat flour. Source: Global Fortification Data Exchange. Chart: Quantity and Proportion of Industrially Processed Food Vehicles. Accessed 04/June/2019. [http://www.fortificationdata.org.]
**Imported rice is assumed 100% industrially produced
Abbreviations: WF, wheat flour; IP, industrially processed; ND, no data

Supplementary Table S1i: Malawi Fourth Integrated Household Survey 2016-2017 (12,447 HH)*

Food	**%**	**Landscape for food****	**Comments**
Salt	99.9	ND	Potential alternative vehicle
Tomato	84.1		
Cooking oil	76.1	ND	Potential alternative vehicle
Nkhwani (pumpkin leaves)	70.7		
Maize ufa mgaiwa (normal flour)	63.9	MF: 15% IP	Maize flour is mandatory
Sugar	56.2	ND	Potential alternative vehicle
Tanaposi/Rape	47.5		
Onion	47.0		
Maize ufa refined (fine flour)	43.7	MF: 15% IP	Maize flour is mandatory
Bean, brown	42.1		
Okra / Therere	40.7		
Sun Dried fish (Small Variety)	40.7		
Mandazi, doughnut (vendor)	34.4	WF: 98% IP	Wheat flour is mandatory
Eggs	31.9		

*As HH sample size was consistent across foods, the individual food sample sizes are not shown.
**The GFDx only collects data for industrially processed maize flour, oil, rice, salt, and wheat flour. Source: Global Fortification Data Exchange. Chart: Quantity and Proportion of Industrially Processed Food Vehicles. Accessed 04/June/2019. [http://www.fortificationdata.org.]
Abbreviations: WF, wheat flour; IP, industrially processed; ND, no data; MF, maize flour

Supplementary Table S1j: Mozambique Household Budget Survey 2008-2009 (Inquérito aos Agregados Familiares)*, N=2,807

Food	**%**	**Landscape for food****	**Comments**
Salt	98.3	ND	Potential alternative vehicle
Water	97.7		
Other cultivated green leafy vegetables	82.7		
Maize ufamgaiwa (normal flour)	68.3	MF: 30% IP	Maize flour is mandatory
Cooking oil	55.1	ND	Potential alternative vehicle
Dried fish	48.6		
Cassava flour	46.7		
Onion	46.4		
Green maize	44.0		Assumed this is fresh maize
Tomato	42.4		
Cowpea (khobwe)	41.8		
Cassava tubers	38.8		
Groundnut	35.7		
Sugar	32.2	ND	Potential alternative vehicle
Bread	31.0	WF: 100% IP	Wheat flour is mandatory

*Several foods with reach >30% or greater removed where sample sizes were 11 or less; all remaining foods had sample sizes of 2,807
**The GFDx only collects data for industrially processed maize flour, oil, rice, salt, and wheat flour. Source: Global Fortification Data Exchange. Chart: Quantity and Proportion of Industrially Processed Food Vehicles. Accessed 04/June/2019. [http://www.fortificationdata.org.]
Abbreviations: WF, wheat flour; IP, industrially processed; ND, no data; MF, maize flour

Supplementary Table S1k: Niger National Survey on Household Living Conditions and Agriculture 2014, Wave 2 Panel Data (l'Enquête Nationale sur les Conditions de Vie des Mênages et l'Agriculture) (1st visit: 3,617 HH, 2nd visit: 3,143 HH)*

Food	**%, 1st visit**	**%, 2nd visit**	**Landscape for food****	**Comments**
Salt	94.2	95.2	ND	Potential alternative vehicle
Maggi cube	93.9	89.2	ND	Potential alternative vehicle
Millet	84.7	81.0	ND	Potential alternative vehicle
Palm oil	80.6	76.4	ND	Potential alternative vehicle
Chilli pepper	77.9	76.5		
Sugar	75.9	75.5	ND	Potential alternative vehicle
Rice	75.9	70.3	ND	Potential alternative vehicle
Fresh onion	73.0	57.2		
Soumbala (base of sorrel or niere)	71.4	67.6		
Dried beans	60.4	51.5		
Okra dry	55.6	41.0		
Maize	55.0	44.3	ND	Potential alternative vehicle
Pasta	52.1	45.4	WF: 100% IP	Wheat flour is mandatory
Baobab leaves	50.4	44.0		
Fresh tomato	49.0	21.2		
Tomato concentrate	47.9	38.5		
Other spices and condiments (garlic, ginger, etc.)	47.7	52.6		
Malahya (Fakkou)	42.4	43.1		
Curdled milk/yogurt (lait caillé)	41.4	45.1		
Cassava flour (attiéke, gari, tapioca)	39.3	22.6		
Dried tomato	37.0	30.2		
Tea in pack or packet	36.7	33.6		
Bean fritters	36.6	36.3		
Cola nuts	35.0	36.3		
Salad (lettuce)	34.9	3.1		
Squash and zucchini	33.4	31.3		
Beef	30.8	22.4		

*As HH sample size was consistent across foods, the individual food sample sizes are not shown.
Foods in red did not have at least 30% reach when reported during the 2nd HH visit of the survey
**The GFDx only collects data for industrially processed maize flour, oil, rice, salt, and wheat flour. Source: Global Fortification Data Exchange. Chart: Quantity and Proportion of Industrially Processed Food Vehicles. Accessed 04/June/2019. [http://www.fortificationdata.org.]
Abbreviations: WF, wheat flour; IP, industrially processed; ND, no data

Supplementary Table S1l: Nigeria - General Household Survey, Panel 2015-2016, Wave 3 (4,580 HH)*

Food	%	Landscape for food**	Comments
Oil and fats: palm oil	92.9	ND	Potential alternative vehicle
Vegetables: onions	90.6		
Salt	89.6	Salt: 20% IP	Potential alternative vehicle
Vegetables: tomatoes	78.0		
Vegetables : fresh pepper	69.9		
Pulses, nuts and seeds, white beans	63.2		
Starchy roots. Tubers & plantain, yam-roots	59.5		
Bread	57.6	WF: 100% IP	Wheat flour is mandatory
Sugar, sweets and confectionary :sugar	52.1	ND	Potential alternative vehicle
Oil and fats: groundnut oil	50.3	ND	Potential alternative vehicle
Grain and flours: rice-local	46.5	Rice: 40% IP	Potential alternative vehicle
Grain and flours: rice-imported	45.6	100% IP***	Potential alternative vehicle
Meat: beef	42.5		
Starchy roots, tubers and plantain: gari-white	37.4		
Fish and seafood: fish-frozen	36.8		
Grain and flours: guinea corn/sorghum	32.9	ND	Potential alternative vehicle
Vegetables: okra-fresh	32.2		

*As HH sample size was consistent across foods, the individual food sample sizes are not shown.
**The GFDx only collects data for industrially processed maize flour, oil, rice, salt, and wheat flour. Source: Global Fortification Data Exchange. Chart: Quantity and Proportion of Industrially Processed Food Vehicles. Accessed 04/June/2019. [http://www.fortificationdata.org.]
***Imported rice is assumed 100% industrially produced
Abbreviations: WF, wheat flour; IP, industrially processed; ND, no data

Supplementary Table S1m: Philippines National Nutrition Survey 2008*

Food	%	Landscape for food**	Comments
Rice	95.2	Rice: 29% IP	Potential alternative vehicle
Sugar	75.3	ND	Potential alternative vehicle
Cooking oil	71.8	ND	Potential alternative vehicle
Coffee, instant	60.8		
Bread	40.6	WF: 100% IP	Potential alternative vehicle
Chicken eggs	33.7		

*Sample sizes for household and foods not reported
**The GFDx only collects data for industrially processed maize flour, oil, rice, salt, and wheat flour. Source: Global Fortification Data Exchange. Chart: Quantity and Proportion of Industrially Processed Food Vehicles. Accessed 04/June/2019. [http://www.fortificationdata.org.]
Abbreviations: WF, wheat flour; IP, industrially processed; ND, no data

Supplementary Table S1n: Sierra Leone Integrated Household Survey 2011

Food	**%**	**n/N**	**Landscape for food***	**Comments**
Salt	93.7	6,299/6,723	ND	Potential alternative vehicle
Onions	89.5	6,016/6,722		
Palm oil	88.5	5,950/6,723	ND	Potential alternative vehicle
Fish-dried	72.3	4,860/6,720		
Rice-imported	69.4	4,668/6,725	100%**	Potential alternative vehicle
Pepper fresh	67.0	4,504/6,723		
Condiments (spices, etc)	64.7	4,341/6,708		
Groundnuts-roasted	58.4	3,921/6,713		
Potato leaves	51.7	3,474/6,717		
Cassava leaves	49.8	3,343/6,710		
Sugar	45.7	3,064/6,710	ND	Potential alternative vehicle
Fish-fresh	45.6	3,063/6,719		
Cassava	38.6	2,592/6,722		
Other foods (not mentioned elsewhere)	37.8	2,533/6,694		
Big beans	35.3	2,369/6,716		
Bread (incl. Rice bread, cassava, bread, etc)	35.0	2,350/6,720	WF: 100% IP	Wheat flour is voluntary
Other leafy vegetables	33.5	2,250/6,713		
Pepper - dried	33.0	2,215/6,713		
Okra-fresh	30.7	2,060/6,719		

*The GFDx only collects data for industrially processed maize flour, oil, rice, salt, and wheat flour. Source: Global Fortification Data Exchange. Chart: Quantity and Proportion of Industrially Processed Food Vehicles. Accessed 04/June/2019. [http://www.fortificationdata.org.]
**Imported rice is assumed 100% industrially produced
Abbreviations: WF, wheat flour; IP, industrially processed; ND, no data

Supplementary Table S1o: Somaliland Household Survey 2013, Adapted for the Somali High Frequency Survey (1,725 HH)*

Food	**%**	**Landscape for food****	**Comments**
Rice, husked	97.3	ND	Potential alternative vehicle
Sugar	95.0	ND	Potential alternative vehicle
Salt	94.7	ND	Potential alternative vehicle
Tea	88.8		
Onion	84.4		
Potatoes	82.8		
Millet, flour	80.6	ND	Potential alternative vehicle
Tomatoes	80.0		
Other cooking oil	78.3	ND	Potential alternative vehicle
Macaroni, spaghetti	72.9	WF: 100% IP	Potential alternative vehicle
Garlic	57.6		
Goat/Sheep	51.4		
Ladies finger/okra	49.6		
Bread	46.4	WF: 100% IP	Potential alternative vehicle
Milk	43.5	ND	Potential alternative vehicle

*As HH sample size was consistent across foods, the individual food sample sizes are not shown.
**The GFDx only collects data for industrially processed maize flour, oil, rice, salt, and wheat flour. Source: Global Fortification Data Exchange. Chart: Quantity and Proportion of Industrially Processed Food Vehicles. Accessed 04/June/2019. [http://www.fortificationdata.org.]
Abbreviations: WF, wheat flour; IP, industrially processed; ND, no data

Supplementary Table S1p: Tajikistan Living Standards Survey 2009 (3,352 HH)*

Food	**%**	**Landscape for food****	**Comments**
Tea	99.6		
Onions	99.1		
Potatoes	98.8		
Salt	98.4	ND	Potential alternative vehicle
Vegetable oil	95.7	ND	Potential alternative vehicle
Sugar	94.7	ND	Potential alternative vehicle
Carrots	90.7		
Rice	86.7	Rice: 100% IP	Potential alternative vehicle
Macaroni products	79.8	WF: 60% IP	Potential alternative vehicle
Flour	76.9	WF: 60% IP	Potential alternative vehicle
Beef	69.0		
Apples	68.6		
Grapes	67.9		
Eggs	63.6		
Fresh milk	59.1	ND	Potential alternative vehicle
Tomatoes	57.3		
Dried beans, pulses (beans, peas, lentils, etc.)	46.0		
Sweets, eastern sweets	40.3		
Butter	37.0		
Pumpkin	34.0		

*As HH sample size was consistent across foods, the individual food sample sizes are not shown.
**The GFDx only collects data for industrially processed maize flour, oil, rice, salt, and wheat flour. Source: Global Fortification Data Exchange. Chart: Quantity and Proportion of Industrially Processed Food Vehicles. Accessed 04/June/2019. [http://www.fortificationdata.org.]
Abbreviations: WF, wheat flour; IP, industrially processed; ND, no data

Supplementary Table S1q: Tanzania National Panel Survey 2014-2015, Wave 4 (3,352 HH)*

Food	**%**	**Landscape for food****	**Comments**
Salt	97.3	ND	Potential alternative vehicle
Onions, tomatoes, carrots and green pepper, other viungo	90.3		
Cooking oil	86.4	ND	Potential alternative vehicle
Maize (flour)	82.3	ND	Maize flour is mandatory
Spinach, cabbage and other green vegetables	77.5		
Sugar	77.3	ND	Potential alternative vehicle
Peas, beans, lentils and other pulses	70.8		
Rice (husked)	66.4	ND	Potential alternative vehicle
Tea dry	64.4		
Fresh fish and seafood (including dagaa)	57.9		
Beef including minced sausage	39.4		
Mangoes, avocadoes and other fruits	34.5		
Irish potatoes	34.5		
Buns, cakes and biscuits	34.2	WF: 98% IP	Wheat flour is mandatory
Coconuts (mature/immature)	32.7		
Cooking bananas, plantains	30.3		

*As HH sample size was consistent across foods, the individual food sample sizes are not shown.
**The GFDx only collects data for industrially processed maize flour, oil, rice, salt, and wheat flour. Source: Global Fortification Data Exchange. Chart: Quantity and Proportion of Industrially Processed Food Vehicles. Accessed 04/June/2019. [http://www.fortificationdata.org.]
Abbreviations: WF, wheat flour; IP, industrially processed; ND, no data

Supplementary Table S2: Surveys where datasets were not accessible but verified to have consumption or expenditure data to describe reach

Country	Survey name, year	Type of data
Cambodia	Cambodia Socio-Economic Survey 2014	AC/E
Cameroon	Cameroon Household Survey 2014<br>(Troisième Enquête Camerounaise auprès des Ménages)	AC/E
Côte d'Ivoire	Household Living Standards Survey 2015<br>(Enquete Niveau de Vie des Ménages 2015)	AC/E
Djibouti	Djibouti Family Health Survey 2012 (PAPFAM)<br>(Deuxieme Enquete Djiboutienne sur la Sante de la Famille PAPFAM 2012)	AC/E
eSwatini	Swaziland Household Income and Expenditure Survey 2009-2010	AC/E
eSwatini	Swaziland National Nutrition Survey 2008	C
Indonesia	Indonesia National Socioeconomic Survey 2017 (SUSENAS)<br>Survei Sosial Ekonomi Nasional (SUSENAS)	AC/E
Indonesia	Total Diet Survey 2015	C
Kenya	Kenya National Micronutrient Survey 2011	C
Laos	Laos Expenditure and Consumption Survey 2007-2008	AC/E
Lesotho	Household Budget Survey 2002-2003	AC/E
Liberia	Liberia Comprehensive Food Security and Nutrition Survey (CFSNS) 2013	AC/E
Mauritania	Mauritania Continuous Household Living Conditions Survey 2014<br>(Enquete permanente sur les conditions de vie des Ménages 2004 (EPCV))	AC/E
Micronesia, Federated States of	Micronesia Household Income and Expenditure Survey 2013-2014	AC/E
Mongolia	Mongolia National Nutrition Survey 2017	C
Morocco	Morocco National Household Consumption and Expenditure Survey 2013-2014<br>(Enquête nationale sur la consommation et les dépenses des ménages)	AC/E
Nepal	Nepal Living Standards Measurement Survey 2010-2011	AC/E
Nepal	Nepal Micronutrient Status Survey 2016	C
Nicaragua	Nicaragua Living Standards Measurement Survey 2014<br>(Encuesta Nacional de Hogares sobre Medición de Nivel de Vida 2014)	
Nigeria	Nigeria Food Consumption and Nutrition Survey 2001-2003	C
Pakistan	Pakistan National Nutrition Survey 2011	C
Pakistan	Pakistan Integrated Household Survey 2001-2002	AC/E
Philippines	National Nutrition Survey 2013	C
Sierra Leone	Sierra Leone National Nutrition Survey 2017	C
Somalia	Somalia National Micronutrient and Anthropometric Survey 2009	C
Sri Lanka	Household Income and Expenditure Survey 2016	AC/E
Syrian, Arab Republic	Household Income and Expenditure Survey 2008-2009	AC/E
Tajikistan	Tajikistan Micronutrient Status Survey 2016	C
Timor-Leste	Timor-Leste Food and Nutrition Survey 2013	C
Timor-Leste	Household Income and Expenditure Survey 2011	AC/E
Uzbekistan	Household Budget Survey 2003	AC/E
Vanuatu	Vanuatu Household Income and Expenditure Survey 2010	AC/E
Vietnam	Vietnam Living Standards Measurement Survey 2012	AC/E
Vietnam	Vietnam General Nutrition Survey 2009-2010	C
Yemen	Yemen Household Budget Survey 2005-2006	AC/E
Zambia	2015 Living Conditions Monitoring Survey (LCMS)	AC/E
Zambia	Food Consumption and Micronutrient Status Report 2014	C
Zimbabwe	Zimbabwe National Nutrition 2018	AC/E

Type of data: AC/E, Apparent Consumption if consumption is collected through an income and expenditure survey; C, if consumption is collected using a dietary intake assessment tool

Supplementary Table S3: LILMIC countries where consumption or expenditure data could not be confirmed (no report or survey tools to evaluate

Country	Survey name, year
Angola	Angola National Nutrition Survey 2007<br>(Relatorio do Inquerito sobre a Nutrição em Angola 2007)
Comoros	Comoros Integrated Household Survey 2004<br>(Enquete Intégrale auprés des Ménages 2004)
Georgia	Georgia Living Conditions, Lifestyles and Health Study 2001-2002
Honduras	Honduras Multipurpose Household Survey, September 2006
Kyrgyzstan	Kyrgyzstan Living Conditions, Lifestyles and Health Study 2001-2002
Kyrgyzstan	Integrated Household Survey 2015
Lesotho	Lesotho Household Survey 2005
Lesotho	Lesotho National Nutrition Survey 2007
Liberia	Liberia National Nutrition Survey 1999-2000
Moldova	Moldova Living Conditions, Lifestyles and Health Study 2001-2002
Mozambique	Mozambique Micronutrient Survey 2012-2013
Myanmar	Household Income and Expenditure Survey 2006
Myanmar	Myanmar National Nutrition Survey 1997
Sao Tome and Principe	2000 Housing Conditions Survey<br>(Pesquisa de Condições Habitacionais de São Tomé e Príncipe 2000)
Tunisia	National Household Survey
Ukraine	Ukraine Living Conditions, Lifestyles and Health Study 2001-2002
Ukraine	Ukraine National Micronutrient Survey 2002
Zimbabwe	Zimbabwe National Micronutrient Survey 1999

Supplementary Table S4: LILMIC countries with no surveys that may contain food consumption or expenditure data

Country	
Burkina Faso	Kiribati
Cabo Verde	Korea, Dem. People's Rep.
Central African Republic	Moldova
Chad	Papua New Guinea
Congo, Dem. Rep.	Rwanda
Congo, Rep.	South Sudan
Eritrea	Sudan
Guinea	Togo
Guinea-Bissau	
Haiti	
Honduras	
Kiribati	

Supplementary Table S1: Studies by food category and outcomes: Dairy products (n=10)

**Efficacy outcomes**				
Reference	*Food details*	*FA dose, meal, study population*	*Outcomes measured*	*Results*
Kelly et al., 1997	Milk (low-fat)	• 200 µg/200 mL milk (single meal) + folic acid-free meal<br>• 16 elderly patients (63-80 y) (no control/placebo)	• Unmetabolized serum folic acid<br>• Serum folate<br>Pre- and 2.25 hr post-meal	• Unmetabolized serum folic acid: no folic acid found in any subjects post-meal<br>• Serum folate: significant increase after meal
Keane et al., 1998	Milk	• 38 µg/100 mL milk, daily; 6 mos<br>• FA: 49 patients (84 y, mean) from Saint James Hospital<br>• Control: 40 patients, two other institutions (81.9 y, mean)	• Serum folate<br>• RBC folate	• Serum folate* (mean, 95% CI):<br>FA: 5.81 (1.1– 17.6) µg/L<br>Control: 2.16 (0.5–9.4) µg/L<br>• RBC folate*(mean, 95% CI):<br>FA: 316.53 (130–905) µg/L<br>Control: 196.11 (95–490) µg/L
Green et al., 2005	Milk powder (*Anmum*™ brand)	• 375 µg/75g of milk powder daily (as 37.5g powder in 200ml water at morning and evening).<br>• 73 female volunteers (36 FA, 37 control); 18-45 y, range	• Plasma folate<br>• Whole blood folate<br>• RBC folate<br>• Plasma total homocysteine<br>• Vitamin B12	• Plasma folate* (mean Δ, 95% CI):<br>Δ FA/control: 35 (30-41) nmol/L<br>• RBC folate* (mean Δ, 95% CI)<br>Δ FA/control: 539 (436-641) nmol/L<br>• tHcy* (geo. mean, 95% CI):<br>Ratio FA/control: 0.86 (0.79-0.94) µmol/L
de Jong et al., 2005	Milk (semi-skimmed; pasteurized and ultra-heat treated (UHT))	• 200 µg/500 mL of pasteurized (p) or UHT milk daily for 4 wks<br>• 69 volunteers (35 FA, 34 control); 18-49 y, range	• Serum folate<br>• RBC folate<br>• Plasma homocysteine	• Serum folate* (mean Δ, 95% CI):<br>Δ UHT+FA/control: 7.0 (4.8-9.2) nmol/L<br>Δ p+FA/control: 6.6 (5.1-8.1) nmol/L<br>• RBC folate* (mean Δ, 95% CI):<br>Δ UHT+FA/control: 32 (10-55) nmol/L<br>Δ p+FA/control: 36 (20-52) nmol/L<br>• tHcy* (mean Δ ± SD):<br>Δ UHT+FA/control: -0.6 ± 1.0 µmol/L<br>Δ p+FA/control: -0.6 ± 0.6 µmol/L
**Food characteristics, sensory, and/or retention outcomes**				
Reference	*Food details*	*FA dose, point of fortification, storage length, co-fortification*	*Outcomes measured, storage and temperature*	*Results*
Achanta et al., 2007	Milk (2%, pasteurized or non-pasteurized)	100 µg, 200 µg, 300 µg, 400 µg (volume not stated)	• Folic acid retention: 1, 7, 14, 21 d<br>• Color (L*a*b*)<br>• pH, viscosity, fat, protein<br>• 9-point hedonic scale (1 = dislike extremely, 5 = neither dislike nor like, 9=like extremely) on overall acceptability, appearance, color, flavor, texture/mouthfeel	• No significant losses after 21 d<br>• No significant effect on L* or a* values, but significant effect on b* values as folic acid levels increased (increasing yellowness)<br>• No significant effect of folic acid on pH, viscosity, fat, or protein<br>• No significant difference in acceptability scores in fortified milk before or after pasteurization. Mean flavor, appearance, and texture/mouthfeel scores were 7.75 or above, indicating consumer acceptance.

Aryana, 2003	Yogurt (fat-free, sugar-free, plain)	0 µg /cup, 75 µg/cup, or 150 µg/cup:<br>• before pasteurization<br>• after pasteurization<br>• evaluated 4 days after manufacturing<br>• 3 replications conducted	• Color (L*a*b* and L*C*h), pH, titratable acidity (TA), viscosity<br>• Flavor and texture evaluation contest	• Lightness (*L**), yellow (*b**), and redness (*a**) increased after fortification<br>• Chroma (*C**) increased and hue angles (*h**) closer to yellow after fortification<br>• pH and TA not affected by fortification<br>• Significant reduction in flavor scores (150 µg/cup)<br>• Significantly lower sensory body and texture scores after fortification (weak body, graininess)<br>• Lower viscosity/increased whey release (150 µg/cup) after pasteurization
Boeneke et al., 2007	Yogurt (fat-free, lemon flavored)	0 µg/224 mL, 167 µg/224 mL, 335 µg/224 mL, 503 µg/224 mL, 670 µg/224 mL:<br>• before pasteurization<br>• after pasteurization<br>• 3 replications conducted	• Folic acid retention at weeks 1, 5<br>• Protein, moisture, ash, fat at week 1<br>• Viscosity, pH, syneresis, L*a*b*, and flavor and texture at weeks 1, 3, 5	• No significant difference in retention due to pasteurization<br>• Lower folic acid retention at higher addition levels at 5 wks<br>• No differences in protein, fat, moisture, ash, viscosity, syneresis, body and texture scores, lightness (*L**)<br>• Significant differences in redness (*a**) and yellowness (*b**) after fortification<br>• Significant decreasing flavor scores after fortification
Perez-Esteve et al., 2016	Yogurt (low fat and full fat; stirred)	360 µg/125 g	L*a*b, and rheological properties at day 0, 7, 21. Samples stored at 4°C in between analysis	Significant effects of fortification on color (L*, a*, b*, C*, h*) but none on pH, syneresis, any rheological properties. Interactions between fortification and storage with color (b*, h*)
Gaur et al., 2018	Traditional Indian Yogurt-Based Drink (*chhansh*)	27 µg/250 mL<br><br>Co-fortified with vitamin A, vitamin D, iron (ferric pyrophosphate), zinc, iodine	Measured at days 1, 2, 3, 6 and 4°, 23°, 40°C:<br>• pH, titratable acidity, color (ΔE as measured by L*a*b*), viscosity<br>• Triangle sensory test: students, mothers with young children	• No effect on pH or TA due to fortification<br>• Significantly different ΔE (perceptible by human eyes) after fortification<br>• No effect on viscosity due to fortification after six days of storage<br>• Students and mothers with young children unable to differentiate fortified samples from controls in color, smell, or taste
N. de Jong et al., 2000	Vanilla custard, vanilla-mixed fruit quark, strawberry yogurt, vanilla-apple yogurt	130 µg/100 g of dairy product<br>Co-fortified with B1, B2, B6, B12, C, D, E, zinc, iodine, calcium, iron, magnesium	Folic acid retention at day 1 and day 14, samples stored at 4-5°C during days 1-13	No significant losses related to storage over 13 days

Supplementary Table S6: ***Studies by food category and outcomes: Condiments/spread (n=7)***

**Efficacy outcomes**				
Reference	*Food details*	*FA dose, meal, study population*	*Outcomes measured*	*Results*
Pentieva et al., 2003	Low-fat spread (produced by Unilever, which tested the product for shelf-life stability up to 3 months)	200 μg/20 g with a low-folate breakfast; followed by morning snack, lunch, and afternoon snack (single meal)<br>13 male volunteers, 31.6 ± 12.2 y (crossover study design)	Plasma folate at 0.5, 1, 1.5, 2, 2.5, 3, 5, 7 and 10h post-meal	Plasma folate concentrations significantly increased for up to 7 h after consumption. However, FA delivered through a spread was less effective than through a tablet: the area under the plasma folate response curve was 67% of an equivalent FA dose in tablet form
**Food characteristics, sensory, and/or retention outcomes**				
Reference	*Food details*	*FA dose, point of fortification, storage length, co-fortification*	*Outcomes measured, storage and temperature*	*Results*
Vinodkumar et al., 2009b	Salt	50 μg/10 g/day, through regular dietary intake in residential school meals by school children for 1 yr<br><br>Co-fortified with vitamin A, B1, B2, B3, B6, B12, calcium, iron, iodine	• Folic acid retention after storage at 30°C and 45% humidity for 6 months Measurements taken at 0, 2, 4 and 6 mos<br>• Folic acid retention after cooking: salt added to sambar (lentil soup), cooked for 20 min<br>• Salt color	• 0.6% loss in folic acid after 6 mos<br>• 0.2% loss in folic acid after cooking<br>• Without vitamin B2, the salt was almost as white as the color of raw salt. However, when vitamin B2 was added, buff-colored specks were seen in the salt<br><br>*Efficacy outcomes and results not reported here due to co-fortification with nutrients. Blood folate not measured
Vinodkumar et al., 2009a	Salt	100 μg/10g/day, through regular dietary intake in residential school meals by school children for 9 mos<br><br>Co-fortified with vitamin A, B1, B2, B3, B6, B12, iron, iodine, zinc	Folic acid retention after storage at 30°C and 45% humidity for 10 months	• 8.12% loss in folic acid after 10 mos<br><br>*Efficacy outcomes and results not reported here due to co-fortification with nutrients. Blood folate (serum) measured
Sangakkara, 2011	Salt	90 mg/kg; refined salt (buffer solutions at 9 or 10 pH); coarse, impure (grey-brown) salt (buffer solution at 9 pH)<br><br>Co-fortified with iodine	• Color (visual observation)<br>• Folic acid retention in dark storage, 45°C, 60% humidity: 6 mos (refined) or 4 mos (coarse)	"The appearance of the salt turns into a pale yellow upon spraying the solutions"<br><br>• 6 mos, refined salt: 95% retention<br>• 4 mos, coarse salt: 95% retention
Li et al., 2011	Salt	20 mg/kg; several samples created with differing technology:<br>• Direct powder blending<br>• Spray as aqueous solution	• Folic acid retention after storage at 45°C and 60% humidity, measured at 0, 1, 2, 3, and 9 mos	Salt: Retention across samples ranged from 80.9%-95.7% after 9 mos<br><br>Sugar: Retention across samples ranged from 50.3%-101.6% after 9 mos

	Sugar	• Premix extrusion and blending<br>3 samples: no co-fortification, co-fortified with iodine, co-fortified with iron + iodine<br><br>20 mg/kg; several samples created with differing technology:<br>• Direct powder blending<br>• Spray as aqueous solution<br>• Premix extrusion and blending<br>• Spray as oil suspension<br>3 samples: no co-fortification, co-fortified with vitamin A, co-fortified with iron + vitamin A	• Color and physical properties during 9 mos storage (visual observation)	"Most salt and sugar samples were not visibly changed in appearance when stored at high temperature and relative humidity.<br><br>When the samples were prepared by spraying the same amount of folic acid solution, the sugar sample was more likely to stick together than the salt sample.<br><br>Folic acid premixes with matching particle sizes to sugar and salt grains remained essentially visibly indistinguishable in the systems, even after 9 months of storage."
McGee, 2012	Salt	30 mg/kg<br><br>Several samples created: no co-fortification (FA only), co-fortified with iodine, iron (encapsulated or not), or iodine and iron (encapsulated or not)	Folic acid retention after 1 year of storage	FA only: 105% ± 6%<br>FA+iodine: 101% ± 6%<br>FA+iodine+iron: ~68% (graph estimate)<br>FA+iodine+encapsulated iron: 76%± 7%<br>FA+iron: ~55% (graph estimate)<br>FA+encapsulated iron: ~63% (graph estimate)
McGee et al., 2017	Salt	3 sets of samples (multiple sample types within a set, with differing weight per volume amounts of micronutrients, buffer solutions, and/or salt origins [Canada or Orissa, India]):<br><br>• 10 mg/kg (coarse salt)<br>Co-fortified with iodine<br><br>• 45 mg/kg<br>Co-fortified with iodine<br><br>• 50 mg/kg<br>Co-fortified with iodine	10 mg/kg:<br>• Color analysis (HSV color space) at production<br>45 mg/kg:<br>• Folic acid retention after storage at 45°C and 60% humidity, measured at 0, 4 mos<br>50 mg/kg:<br>• Folic acid retention after storage at ~25°C, measured at 0, 12 mos<br>• Folic acid retention after storage at ~25°C and 45°C, with fluctuating humidity (20%-60%) measured at 0, 20 mos<br>• Color analysis (HSV)	• At production: Uneven yellowing due to uneven folic acid dispersion amongst larger crystals<br><br>• Folic acid retention after 4 mos ranged from 87%-96%<br><br>• Folic acid retention:<br>○ ~25°C,12 mos: >80%<br>○ ~25°C, 20 mos: >60%<br>○ 45°C, 20 mos: >55%<br>• Color analysis:<br>○ 0 mo: pale yellow, uniform (hue=58.0°)<br>○ ~25°C, 20 mos: pale yellow, uniform (hue=49°)<br>○ 45°C, 20 mos, fluctuating humidity: pinkish, uniform (hue=29°)

Supplementary Table S7: Studies by food category and outcomes: Beverages (n=7)

**Food characteristics, sensory, and/or retention outcomes**				
Reference	*Food details*	*FA dose, point of fortification, storage length, co-fortification*	*Outcomes measured, storage and temperature*	*Results*
Öhrvik et al., 2008	Orange juice	0.4 mg/kg<br>0.6 mg/kg<br><br>Co-fortified with iron	Folic acid retention at day 1, 21, 35 (best before date)	No significant differences in folic acid concentrations between day 1, 21, and 35
Rivas et al., 2007	Mixed orange juice (50%) and UHT skim milk (20%) beverage, with water (30%), sugar (7.5%), citric acid (0.1%), pectin (0.3%)	1,283.5 mg/kg<br><br>Co-fortified with vitamins B2, B5, B7	• Folic acid retention using non-thermal preservation and thermal pasteurization (84°C and 95°C at 15–120 seconds)<br>• Folic acid retention at 81 days, 4°C	• No significant differences in folic acid concentrations using non-thermal preservation or using thermal pasteurization at 84°C<br>• At 95°C, folic acid losses ~10% (graph estimate)<br>• Folic acid retention 28%-36% after 15 days, constant for the rest of the storage period.
Ruiz-Rico et al., 2017	Apple juice<br>Orange juice	4 mg encapsulated FA (eFA)/10 mL<br>0.3 mg/10 mL (free FA)	• Folic acid retention under 121°C, 1 bar, for 5, 10, 15 mins<br><br>• Folic acid retention under visible and ultraviolet lamps for 16, 18, 20, 22, 24 h<br><br>• Folic acid retention at 28 days, 4°C	121°C, 1 bar<br>Apple juice:<br>• eFA: 70%-80% retention (graph estimate)<br>• free FA: 58%-66% retention<br>Orange juice:<br>• eFA: 81-91% retention<br>• free FA: 81-85% retention<br>Visible light treatment after 24 hours:<br>Apple juice<br>• eFA: 81-91% retention / free FA: 24% retention<br>Orange juice<br>• eFA: No significant losses<br>• free FA: No significant losses<br>Ultraviolet light treatment after 24 hours:<br>Apple juice<br>• eFA: 44% retention / free FA: 36% retention<br>Orange juice<br>• eFA: 73% retention / free FA: 83% retention<br>28 days, 4°C:<br>Apple juice<br>• eFA: 100% retention / free FA: 77% retention<br>Orange juice<br>• eFA: 100% retention / free FA: 80% retention

Frommherz et al., 2014	Commonly sold vitamin juices (n=9) (no description of juice content)	Label claims:<br>100 µg/100 mL for 8 juices<br>60 µg/100 mL for 1 juice<br><br>Actual folic acid content (average):<br>186.0 ± 33.6 µg//100 mL	• Folic acid retention after 12 mos, 18°C<br>• Folic acid retention after 12 mos, 18°C, 500=lux for 10 h/day	• Average loss of 81 µg/100 ml (46%) over the course of the entire study<br>• Average loss of 87 µg/100 ml (50%) over the course of the entire study<br><br>*Juices were overfortified by on average 89%, such that by 12 mos, juices were still +4% or -8% below the label's nutrient claim
Kida et al., 2018	Acidic beverage (no further description)	3.16 mg/L<br><br>Fortified with varying levels of ethanol (0%, 0.05%, 0.1%, 0.3%, 0.5%, and 1%)	• Folic acid retention at 6 days, 50°C<br>• Analysis of drink ingredient components affecting Folic acid retention at 13 days, 50°C	• 6 days: Folic acid retention declined and plateaued with increasing ethanol levels (90% at 0% ethanol and 55% at 1% ethanol)<br>• 13 days: Flavor #3 affected folic acid retention the most (22.7%), while nicotinamide the least (65.1%)
N. de Jong et al., 2000	Multiple fruit juices: orange-peach juice, apple-berry-grape juice, apple compote, apple-peach compote	130 µg/100 g of dairy product<br><br>Co-fortified with vitamins B1, B2, B6, B12, C, zinc, iodine, calcium, iron, magnesium	• Folic acid retention at day 1 and day 14, samples stored at 4-5°C during days 1-13<br>• Samples measured by two different labs, during different seasons	No significant losses related to storage over 13 days, although nutrient content both at day 1 and day 14 were related to the type of fruit juice
Tapola et al., 2004	Mineral water	563 µg/750 mL<br><br>Co-fortified with vitamin B6, B12, D, calcium	Folic acid retention after 6 mos	60% retention after 6 mos<br><br>*Efficacy outcomes and results not reported here due to co-fortification with nutrients. Serum folate and RBC folate measured

Supplementary Table S8: Studies by food category and outcomes: Meat and eggs (n=6)

**Food characteristics, sensory, and/or retention outcomes**				
Reference	*Food details*	*FA dose, point of fortification, storage length, co-fortification*	*Outcomes measured, storage and temperature*	*Results*
House et al., 2002	Chicken eggs	40-50 µg/egg, fortification through enriched hen feed	Folic acid retention at 4°C, on days 0, 7, 14, 21, 28	No significant nutrient losses during storage
Altic et al., 2016	Chicken eggs	1.23 mg/kg, fortification through enriched hen feed	• Folic acid retention at 4-7°C, on days 1, 7, 14, 21, 17<br>• Folic acid retention at 18-20°C, on days 1, 7, 14, 21, 17<br>• Folic acid retention after cooking via: boiling, poaching, frying, scrambling at 2 time points for each method (50 s or 2 min)	• No significant differences in nutrient content during storage, regardless of temperature<br>• No significant differences in nutrient content during cooking, regardless of method or cooking time
Galán et al., 2010	Hamburgers (fresh beef meat)	6, 12, or 24 mg/kg	• Folic acid retention after meat irradiation treatment<br>• Folic acid retention after cooking<br>• Texture Profile Analysis (TPA): hardness, springiness, cohesiveness, gumminess and chewiness<br>• Shear Analysis: shear force and work of shearing<br>• Color: L*a*b*, hue angle, saturation index<br>• Acceptability, measured via:<br><u>Descriptive</u>: 10-point hedonic scale (0 = dislike extremely and 10 = like extremely) to evaluate raw hamburgers (odor, color and overall acceptability) and of cooked ones (dour, color, texture, taste and over- all acceptability).<br><u>Preferential:</u> method not described	• Irradiation treatment caused a 20-30% decrease in FA<br>• No significant losses in folic acid after cooking<br>• No effect from FA on TPA or Shear Analysis parameters<br>• Significantly lower b* (yellow) values in raw FA hamburgers compared to non-fortified, and increased red color in raw FA hamburgers (decrease in hue angle and increase in saturation index)<br>• No effect from FA on color with cooked hamburgers<br>• FA did not affect any acceptability parameters in the descriptive test or preferential test
Galán et al., 2011b	Fermented dry ready to-eat sausages (reduced-fat *salchichón*)	6, 12, or 24 mg/kg	• Folic acid retention after meat irradiation treatment<br>• Folic acid retention after cooking<br>• Texture Profile Analysis (TPA): hardness, springiness, cohesiveness, gumminess and chewiness	• Irradiation treatment caused a 15-29% decrease in FA<br>• No significant losses in folic acid after cooking<br>• Significantly higher hardness, shear force, and work of shearing values in 1.2

			• Shear Analysis: shear force and work of shearing<br>• Color: L*a*b*, hue angle, saturation index<br>• Acceptability, measured via:<br>Descriptive: 10-point hedonic scale (0 = dislike extremely and 10 = like extremely) to odor, color, texture, taste and overall acceptability<br>Preferential: tasters asked to rank four samples: 1 = worst, 4 = best	mg and 2.4 mg FA sausages compared to control and 0.6 mg FA sausages<br>• L* and a* values not affected by FA, b* values "minimally" affected by FA.<br>• FA did not affect any acceptability parameters in the descriptive test or preferential test
Galán et al., 2011a	Cooked sausages (*mortadella*)	6, 12, or 24 mg/kg	• Folic acid retention after meat irradiation treatment<br>• Folic acid retention after cooking<br>• Texture Profile Analysis (TPA): hardness, springiness, cohesiveness, gumminess and chewiness<br>• Shear Analysis: shear force and work of shearing<br>• Color: L*a*b*, hue angle, saturation index<br>• Acceptability, measured via:<br>Descriptive: 10-point hedonic scale (0 = dislike extremely and 10 = like extremely) to evaluate odor, color, texture, taste, and overall acceptability<br>• Preferential: tasters asked to rank four samples: 1 = worst, 4 = best	• Irradiation treatment caused 30% decrease in FA<br>• No significant losses in folic acid after cooking<br>• No effect from FA on TPA or Shear Analysis parameters<br>• Significantly higher lightness (L*), decrease in redness (a*) and increase in yellowness (b*) in FA sausage.<br>• FA did not affect any acceptability parameters in the descriptive test or preferential test
Galán et al., 2013	Fresh hamburger, cooked sausages (*mortadella*), dry fermented sausages (*salchichón*)	24 mg/kg	• Folic acid retention after irradiation<br>• Folic acid retention in storage for 60, 90 d<br>• Acceptability of meat products, at d 0, 60, and 90 d, using a descriptive test: 10-point hedonic scale (0 = dislike extremely and 10 = like extremely) to evaluate odor, color, texture, and taste	• Irradiation treatment caused 15-30% decrease in FA<br>• No significant losses in folic acid after 90 d in storage, except for non-irradiated *salchichón*, with 20% losses<br>• FA did not affect any acceptability parameters in the descriptive test at 60 or 90 d

Supplementary Table S9: Studies by food category and outcomes: Fruit, cereal grains, candy (n=5)

**Food characteristics, sensory, and/or retention outcomes**				
Reference	*Food details*	*FA dose, point of fortification, storage length, co-fortification*	*Outcomes measured, storage and temperature*	*Results*
Peña C et al., 2013	Cape gooseberry (fresh)	20% of daily recommended value/200 g<br><br>Vacuum-impregnated (VI) with nutrients<br><br>Co-fortified with calcium, vitamins C, D, and E	• Color: L*a*b*, Hue angle, chroma<br>• Texture<br>• Acceptability at 3, 18, and 21 d storage at 4°C:<br>o General appearance, odor, flavor, and texture rated using a hedonic scale from 0 to 5 (0= no perception, 1 is weak, 2 is less weak, 3 is average, 4 is less strong and 5 is strong)<br>o General quality was assessed on a scale of 0 to 3, where 0 is no quality	"The VI process does not significantly affect the physicochemical properties of The VI process produces significant changes in the sensorial perception of the cape gooseberry, mainly by conferring a greater intensity of orange color and superficial stains, less fresh appearance, greater sweetness, less cape gooseberry characteristic taste, softer texture, less firmness and juicy…the general quality of the [fresh gooseberry] exhibited a greater score than the [fortified gooseberry]"
Tapia et al., 2014	Papaya slices (fresh)	60% of daily allowance/160 g<br><br>Vacuum-impregnated (VI) with nutrients<br><br>Co-fortified with vitamin C, zinc and calcium	• Folic acid retention at 4°C, 12 d<br>• Flavor-related parameters: soluble solids, total solids, pH, tritatable acidity, texture<br>• Acceptability, via a hedonic scale 1-7 with trained panelists and consumer's tests	• Stability results not shown<br>• Flavor parameter results not shown, but reported as "kept up during the evaluated period" and "functional fruit product maintained an intense red-orange color and an improved texture as compared to untreated fruit"<br>• Sensory tests with a trained panel showed good acceptance; consumer panels yielded an acceptance >80%
Moreno et al., 2016	Apple slices (dried)	Impregnation solution of 200 mg/L apple juice used to fortify dried apple slices; final folic acid content depended on drying temperature, fortification method (vacuum impregnation or ohmic heating)	• Mechanical properties (maximum force)<br>• Color (L*a*B, ΔE*, brown index, chroma, hue angle)	• Significantly higher firmness in fortified apple slices compared to control.<br>• Significantly higher overall color score (ΔE), indicating visibly different samples compared to controls.

Tripathi et al., 2011	Finger millet flour (*eleucine coracana)*	1.4 mg/1 kg<br><br>Co-fortified with iron	Shelf life parameters: moisture and free fatty acids after 30 and 60 d	No significant changes to moisture or free fatty acids at 30 or 60 d
Pallavi et al., 2014	Indian traditional sweet (peanut *chikki*)	26.6 mg ± 0.668/kg<br><br>Co-fortified with calcium, iron, vitamin A	• Breaking strength/snap<br>• Color (L*a*B, ΔE*)<br>• Moisture<br>• Acceptability for color, snap, hardness, crunchiness, sweetness, peanut flavor and the overall quality score were evaluated by drawing a vertical line on the scale for coded products<br>• Shelf life parameters: moisture, texture and peroxide value during storage at 37 ± 1°C and 27 ± 1°C	• No significant differences in breaking strength, hardness, and crunchiness, and sweetness<br>• Significantly lighter color in fortified chikki compared to control, but overall color scores were not significant.<br>• Fortified chikki and control were similar in all quality attributes<br>• No significant difference moisture during storage up to 105°C

Supplementary Table S2: Efficacy studies including fortification with folic acid but eliminated for not being able to isolate folic acid effect (sorted by food) (n=26)

Reference	Food details	Co-fortificant nutrients*	Outcome measure**
**Milk/dairy**			
Baro et al., 2003	Skimmed milk: *Puleva Omega 3*	Oleic acid and PUFA of the n-3 series, and vitamins A, D, E, B6	Risk factors for CVD in healthy volunteers: fatty acid composition of plasma, plasma lipids, vitamin E, LDL oxidation, plasma levels of ICAM-1, VCAM-1, Hcy, plasma B12, and **plasma folate**
Carrero et al., 2004	Skimmed milk: *Puleva Omega 3*	Oleic acid and PUFA of the n-3 series, and vitamins A, D, E, B6	Risk factors for CVD in mildly hyperlipidemic volunteers: Plasma triacylglycerols, cholesterol, and HDL cholesterol, plasma fatty acid profile, tHcy, plasma vitamin E, **plasma folate**, lipoprotein(a), VCAM-1, and ICAM-1
Carrero et al., 2005	Skimmed milk: *Puleva Omega 3*	Oleic acid and PUFA of the n-3 series, and vitamins A, D, E, B6	Risk factors for CVD and clinical outcomes in male PVD patients: triacylglycerols, total, LDL, and HDL cholesterol, plasma fatty acid profiles, plasma apolipoprotein B, tHcy, vitamin E, malondialdehyde, plasma vitamin B-6, **plasma folate, RBC folate,** plasma vitamin B-12, plasminogen activator inhibitor 1, E-selectin, sVCAM-1, sICAM-1 concentrations, hs-CRP, oxidized LD; Clinical outcomes: PFWD, ABI
Carrero et al., 2006	Skimmed milk: *Puleva Omega 3*	Oleic acid and PUFA of the n-3 series, and vitamins A, D, E, B6	Risk factors for CVD and clinical outcomes in PVD patients: Plasma triacylglycerols, HDL and LDL cholesterol, plasma fatty acid profile, **plasma folate**, tHcy; Clinical outcomes: PFWD, ABI
Carrero et al., 2007	Skimmed milk: *Puleva Omega 3*	Oleic acid and PUFA of the n-3 series, and vitamins A, D, E, B6	Risk factors for CVD in MI patients: triacylglycerols, total, HDL, and LDL cholesterol, plasma fatty acid profile, plasma apolipoprotein B, tHcy, vitamin E, malondialdehyde, plasma vitamin B-6, **plasma and RBC folate**, sVCAM-1, sICAM-1 concentrations, hs-CRP concentrations, oxidized LDL
Fonolla et al., 2009	Skimmed milk: *Puleva Omega 3*	Oleic acid and PUFA of the n-3 series, and vitamins A, D, E, B6	Risk factors for CVD in volunteers with moderate CVD risk: plasma glucose, triacylglycerols, cholesterol, HDL and LDL cholesterol, tHcy, **plasma folate**, hs-CRP
Martin-Bautista et al., 2010	Skimmed milk: *Puleva Omega 3*	Oleic acid and PUFA of the n-3 series, and vitamins A, D, E, B6	Weight, height, BMI, plasma fatty acids, plasma vitamin E, **plasma folate, RBC folate**, plasma B6; bone metabolism: 25(OH)D, serum calcium, type I collagen carboxy-terminal telopeptide and intact parathormone levels, serum osteocalcin, plasma malondialdehyde
Fonolla-Joya et al., 2016	Skimmed milk (low lactose): *Puleva Digestiva*	EPA, DHA, oleic acid, soluble fiber, calcium, phosphate, vitamins B1, B3, B5, B6, B8, C, E	Risk factors for CVD and bone metabolism markers in healthy post-menopausal women: Serum total cholesterol, HDL and LDL cholesterol, triglycerides, and glucose concentrations, tHcy, plasma B6, **plasma folate, RBC folate**, hs-CRP, serum calcium, 25(OH)D; Bone metabolism: intact-parathyroid hormone, bone-specific alkaline phosphatase, b-crosslaps, serum osteoprotegerin, and receptor activator of nuclear factor kB ligand
Baro L, et al. 2004	Dairy product: *Puleva Mama*	Vitamins A, B1, B2, B3, B5, B6, B7, C, D, E, calcium, phosphorus, zinc, magnesium, copper, iron, iodine, choline***	**Plasma folate, RBC folate**, plasma B12, tHcy, lipids, serum transferrin, serum ferritin, plasma vitamin E

Romeo et al., 2011	Dairy product: *Puleva Max*	EPA, DHA, oleic acid, vitamins A B1, B2, B3, B5, B6, B7, B12, C, D, E, calcium, phosphorus, zinc	White blood cells, lipid profile, serum proteins, total serum calcium, 25(OH)D, glucose, insulin, adiponectin and soluble circulating adhesion molecules levels
Benito et al., 2006	Semi-skimmed milk: *COVAP*	Oleic acid and PUFA of the n-3 series, vitamin E	Risk factors for CVD in MS patients: total and HDL cholesterol, apolipoprotein B, triglycerols, glucose, insulin, hs-CRP, Hcy, and fatty acids contents in serum phospholipids
Cheong et al., 2016	Milk powder: *Anmum Materna*	Vitamins B6, B12, D	**Plasma folate, whole blood folate**, serum B12, 25(OH)D, and plasma pyridoxal 5'-phosphate
Grieger et al., 2009	Whole milk	Calcium, vitamin D	Nutrient intake, serum calcium, 25(OH)D, **serum folate**, vitamin B12, C-terminal collagen I telopeptide, and terminal propeptide of type I procollagen, parathyroid hormone, bone turnover, quantitative heel ultrasound, falls, timed up and go, speed, and hand grip strength
Achón et al., 2011	Whole milk<br>Skimmed milk	Vitamins A, D, E, calcium and phosphorus	**Plasma folate**
Ntzouvani et al., 2013	Low-fat milk	Plant sterols, linoleic acid, alpha linolenic acid, vitamins A, B6, B12, C, E, magnesium, selenium	Enzymatic activity of lyso-PAF AT and Lp-PLA2 in leukocytes and serum, fasting triacylglycerol, HDL and LDL cholesterol, triglycerides, glucose, apolipoprotein A1, apolipoprotein B, insulin, tHcy, **plasma folate**, vitamin B12, total antioxidant capacity, CRP, interleukin-6, tumour necrosis factor alpha
Petrogianni et al., 2014	Low-fat milk	Plant sterols, linoleic acid, alpha linolenic acid, vitamins A, B6, B12, C, E, magnesium, selenium	Weight, height, blood pressure, fasting HDL cholesterol, LDL cholesterol, very-LDL cholesterol, tHcy, **plasma folate**, vitamin B12, fatty acids content in RBC membranes
Kuriyan et al., 2016	Malt and cocoa-based milk	Vitamins A, C, D, B1, B2, B3, B6, B7, B12, copper, iodine, iron, manganese, phosphorus, selenium, zinc	Hemoglobin, serum ferritin, soluble transferrin receptor, CRP, vitamin B2, B12, D, **RBC folate**, serum selenium, morbidity: diarrhea, vomiting, fever, runny nose, visit to physician, hospitalizations; physical performance, cognitive tests, height, weight
Mardones et al., 2008	Milk powder: *Maman*	Omega 3 and omega 6 fatty acids, selenium, iron, zinc, manganese, calcium, phosphorus, vitamin A, B1, B2, B3, B6, B7, C	Birth weight, gestation duration, infant length, head circumference
Wibowo et al., 2016	Milk powder	Calcium, zinc, vitamins A, B1, C, DHA, prebiotic (inulin), and *B. animalis* subsp. *lactis* DR10	Hemoglobin, transferrin, complete blood count, serum ferritin, retinol, 25(OH)D, vitamins B1, B2, B12, zinc, phospholipid, **plasma folate**; DHA, *B. lactis* HNO19 in fecal samples, tumor necrosis factor-α, interleukin-6, polymorphonuclear leukocyte phagocytosis; birth outcomes (delivery date, mode, birth weight, and APGAR score)
**Other beverages**			
Järvenpää et al., 2007	Mineral water	Calcium, vitamins B6, B12, D	**Serum folate, RBC folate,** serum vitamin B12, Plasma Hcy

Mayengbam et al., 2019	5 Hour Energy™, decaffeinated	Vitamins B6, B12	Glucose metabolism, incretin response, metabolomics profile, responsiveness to a glucose load
Carter et al., 2010	Beverage (no details)	Vitamins A, C	Plasma vitamin C, **serum folate**
Oliva et al., 2016	Beverage (no details)	Vitamin B2, B12, D	Vitamin D, B12, B2, **blood folate** (not specified)
**Condiments**			
Kumar et al., 2007	Salt	Vitamins A, B1, B2, B3, B6, B12, calcium, iron, iodine	Hemoglobin, hematocrit, RBC count, urinary iodine, serum vitamin A, cognitive tests for memory, concentration, IQ
Kumar et al., 2014	Salt	Vitamin A, B12, iron, iodine	Hemoglobin, serum retinol, urinary iodine, CRP, AGP
Chen et al., 2011	Seasoning powder	Powder I: vitamin A<br>Powder II: vitamin A and iron<br>Powder III: vitamins A, B1, B2, B3, B9, iron, zinc, calcium	Serum retinol, serum ferritin, weight, height, morbidity (respiratory outcomes, diarrhea outcomes)

*In addition to folic acid

Blood folate outcomes are **bolded

***Not specified which nutrients are extrinsic to the dairy product

Abbreviations (in order of appearance): PUFA, polyunsaturated fatty acids of the n-3 series; CVD, cardiovascular disease; LDL, low-density lipoprotein; ICAM-1, intercellular adhesion molecule-1; VCAM-1, Vascular cell adhesion molecule-1; tHcy, total fasting homocysteine concentrations; Hcy, homocysteine (not described as total fasting); HDL, high density lipoprotein; PVD, peripheral vascular disease; sVCAM-1, soluble vascular adhesion molecule 1; sICAM-1, soluble intercellular cell adhesion molecule 1; hs-CRP, high-sensitivity C-reactive protein; PFWD, pain-free walking distance; ABI, ankle-brachial index; MI, myocardial infarction; BMI, body mass index; 25(OH)D, plasma 25 hydroxyvitamin D; EPA, eicosapentaenoic acid; DHA, docosahexaenoic acid; MS, multiple sclerosis; APGAR; Appearance, Pulse, Grimace, Activity, and Respiration; IQ, intelligence quotient; AGP, alpha(1)-acid glycoprotein
